# Supplementary material for: Cellular profiling of a recently-evolved social behavior in cichlid fishes
Source: Nat Commun. 2023 Aug 14;14:4891. doi: 10.1038/s41467-023-40331-9 (PMC10425353; doi:10.1038/s41467-023-40331-9)
Supplement: Supplementary file 1 — Supplementary Information [file 41467_2023_40331_MOESM1_ESM.docx]

**SUPPLEMENT**

**SUPPLEMENTARY FIGURES**


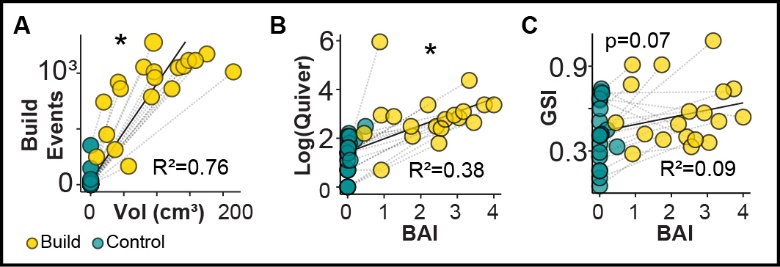


**Figure S1. Building, quivering, and relative gonadal mass are positively correlated across test subjects.** (A) Structural change measured through depth sensing (adjusted for body size) was positively correlated with building behaviors predicted through action recognition (Pearson’s correlation, two-sided, p=8.15x10^-13^), and these measures were combined into a single Bower Activity Index (BAI, x-axis in B and C). BAI was positively correlated with quivering behaviors (B, Pearson’s correlation, two-sided, p=3.35x10^-5^), and trended toward a positive correlation with GSI (C, Pearson’s correlation, two-sided, p=0.07). In all plots, gray lines link paired building/control males.

**
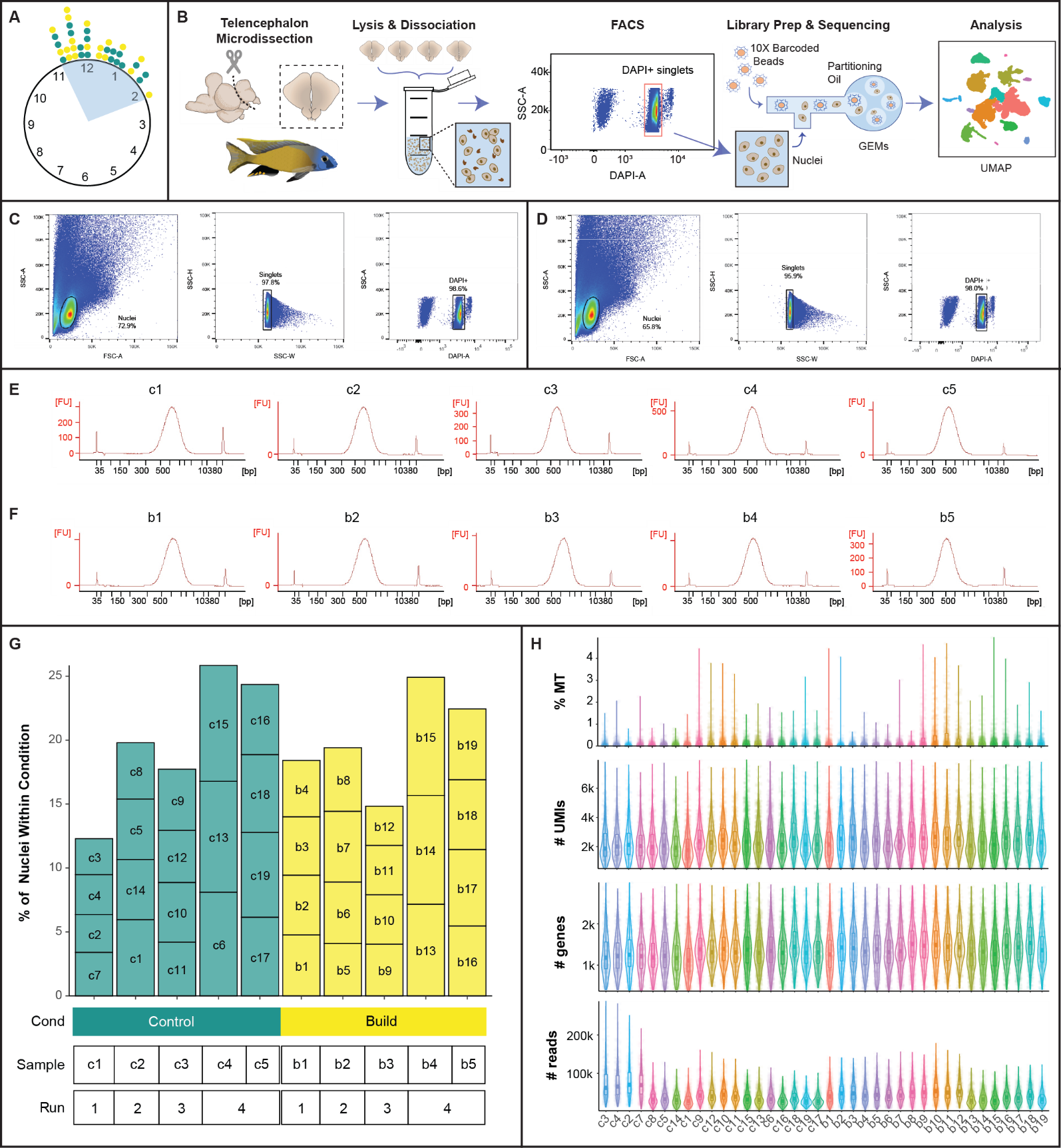
**

**Figure S2. Single nuclei sampling and library metrics.** (A) 19 pairs of building (yellow) and control (turquoise) males were collected at the same time on the same day between 11:30A-230P EST. (B) Schematic of experimental pipeline for snRNA-seq. (C-D) Two example FACS gating sequences for isolating single nuclei based on forward and side scatter area, side scatter height and width, and DAPI fluorescence. (E-F) Library size distributions for all ten sample pools. (G) Proportion of nuclei sampled by condition, sampling pool, nuclei isolation run (each run was a different day), and subject (partitions within each bar). (H) Sequence metrics across nuclei for each test subject (x-axis), including percentage of genes that were mitochondrial (top panel), total number of Unique Molecular Identifiers (UMIs, second panel), total number of unique genes (third panel), and total number of reads (bottom panel); n=33,674 individual nuclei sampled across 38 biologically independent animals. In box plots, the center line indicates the median, the bounds of the box indicate the upper and lower quartiles, and whiskers indicate 1.5x interquartile range. Source data are provided as a Source Data file. Fish artwork in panel B is reprinted from iScience, Vol 23 / Issue 10, Lijiang Long, Zachary V. Johnson, Junyu Li, Tucker J. Lancaster, Vineeth Aljapur, Jeffrey T. Streelman, Patrick T. McGrath, Automatic Classification of Cichlid Behaviors Using 3D Convolutional Residual Networks, 2020, with permission from Elsevier.


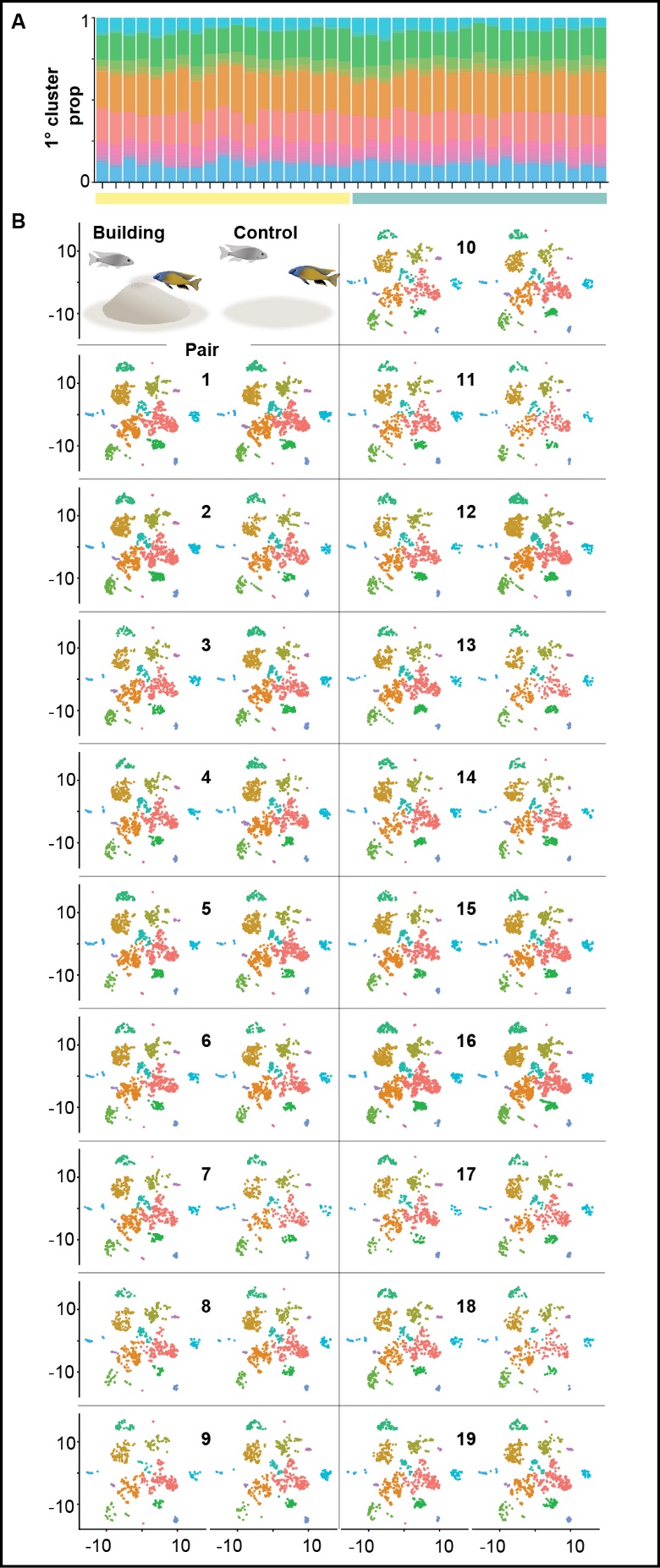


**Figure S3. Cluster composition is consistent across individuals.** (A) Stacked bar chart showing consistent sampling of clusters across test subjects (yellow = build, turquoise = control). (B) Individual UMAP plots of nuclei sampled from all 38 test subjects, organized into 19 pairs with building subjects on the left and paired control subjects on the right. All except the two smallest primary clusters (3_Peri, 7_GABA) were sampled from every individual male. Fish artwork in panel B is reprinted from iScience, Vol 23 / Issue 10, Lijiang Long, Zachary V. Johnson, Junyu Li, Tucker J. Lancaster, Vineeth Aljapur, Jeffrey T. Streelman, Patrick T. McGrath, Automatic Classification of Cichlid Behaviors Using 3D Convolutional Residual Networks, 2020, with permission from Elsevier.


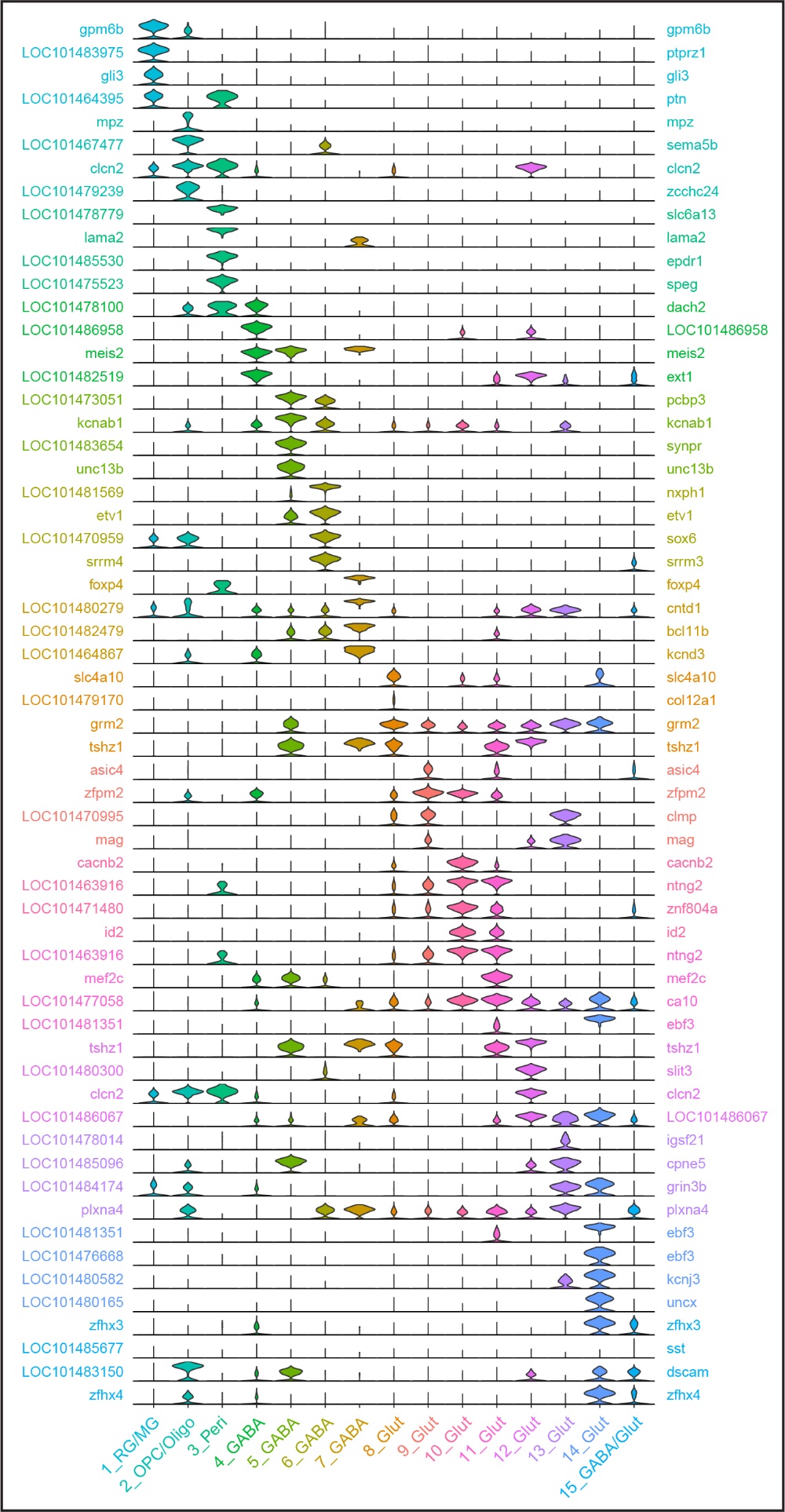


**Figure S4. Top marker genes by cluster.** Violin plots of the top four marker genes for each 1° cluster. Cichlid gene ID’s are shown on the left and common names of predicted gene homologs are shown on the right.


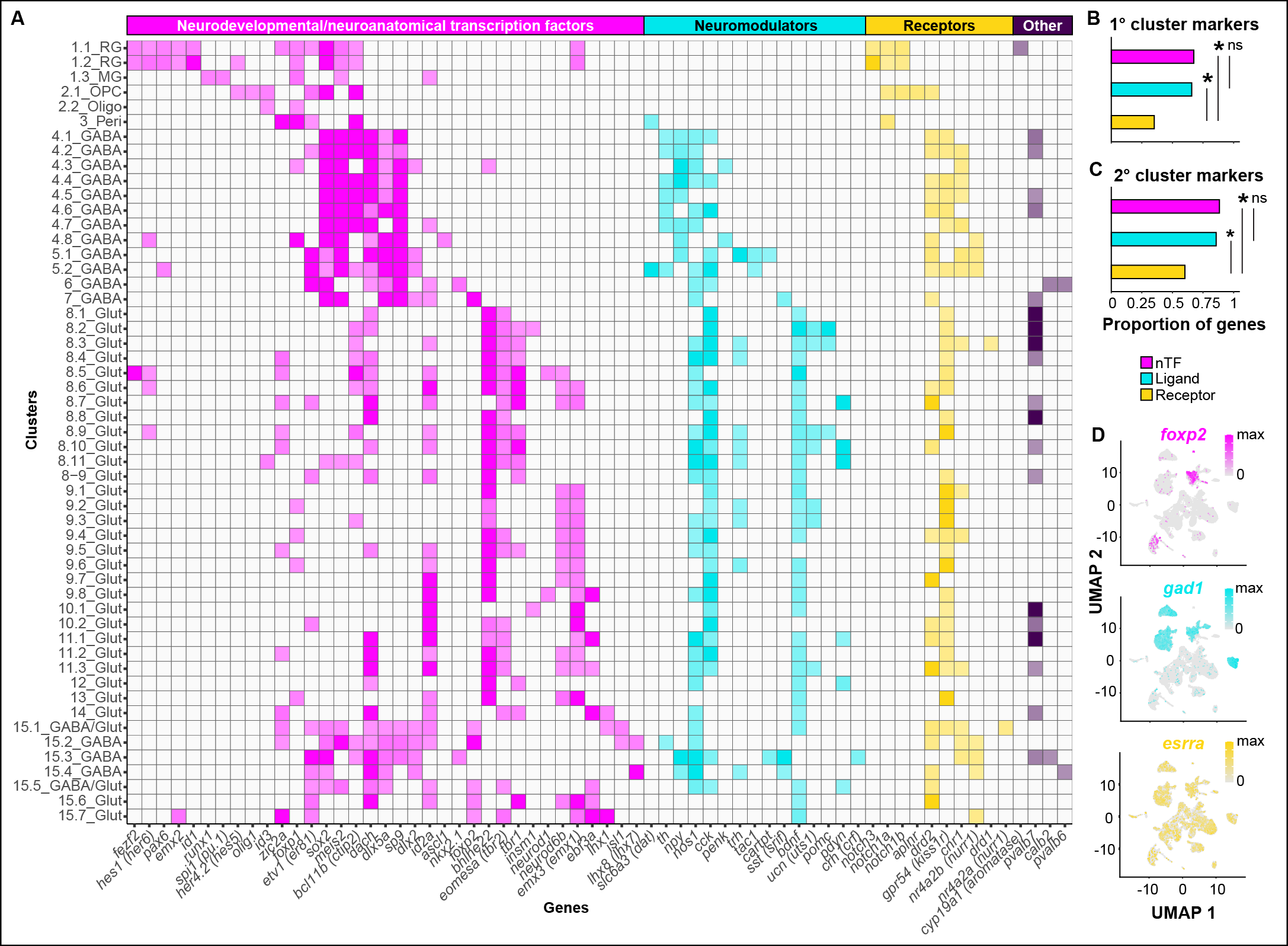


**Figure S5. Clusters have unique molecular barcodes of nTF, ligand, and receptor genes.** (A) Expression profiles of genes encoding nTFs (pink), ligands and related transporters (light blue), and receptors (yellow) across 1° and 2° clusters. Each cluster is distinguished by a unique barcode of anatomical markers and signaling molecules. (B, C) 1° and 2° cluster marker genes are differentially enriched for genes encoding nTFs and ligands compared to receptors (Fisher’s Exact Tests, two-sided). (D) Example genes that reflect these category-specific trends (color-coded to match categories in B, C).


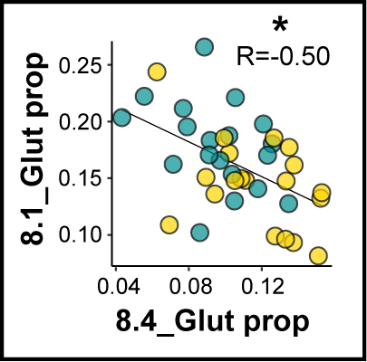


**Figure S6. Neuronal rebalancing of 8.4_Glut and 8.1_Glut.** The relative proportions of 8.4_Glut and 8.1_Glut is negatively correlated across individuals (Pearson’s R correlation, two-sided, p=0.0012).


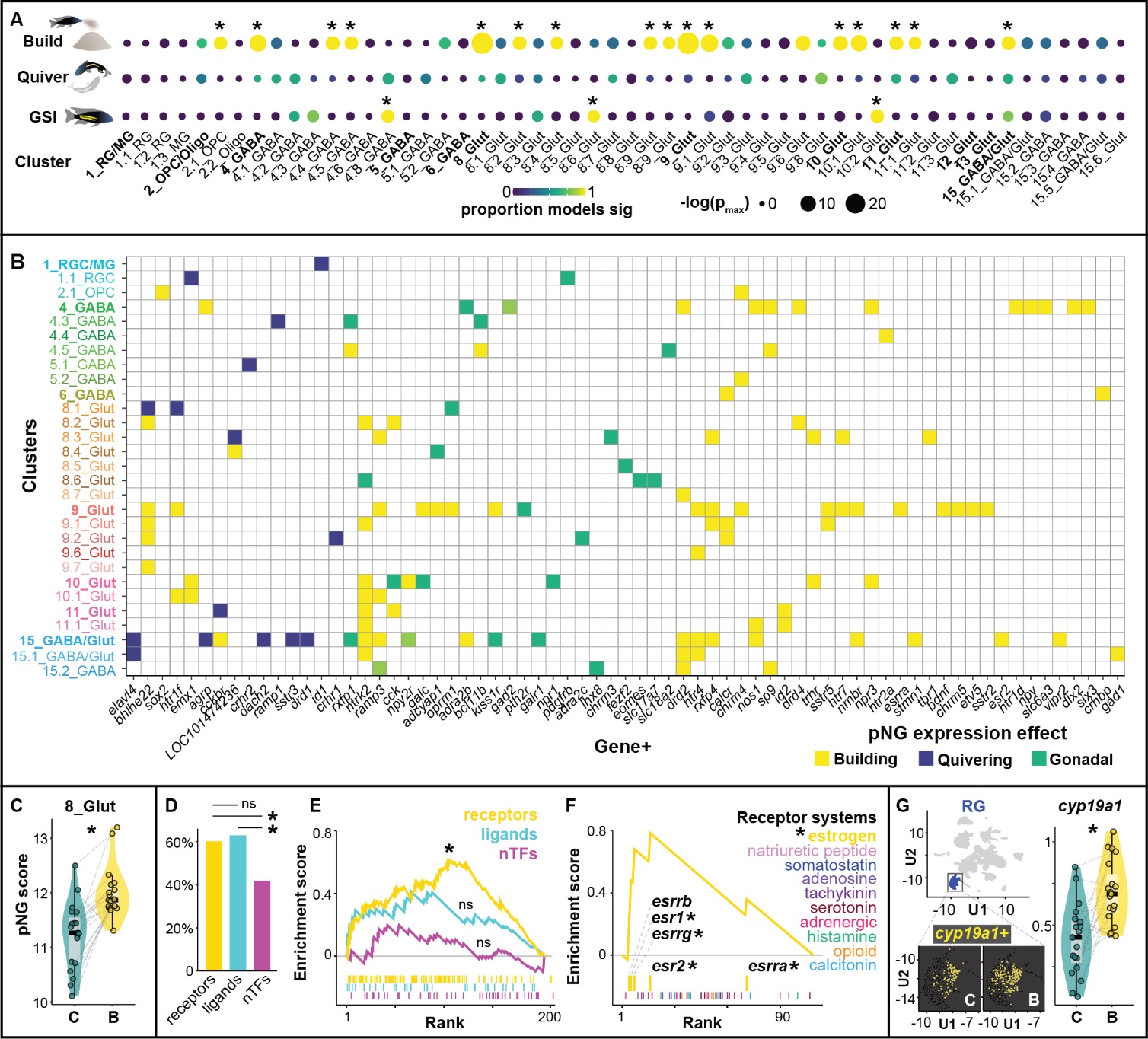


**Figure S7. Behavior and gonadal physiology are associated with transcriptional signatures of neurogenesis in distinct cell populations.** (A) Bower construction, but not quivering behavior, is associated with increased pNG expression in a large set of 1° and 2° clusters, whereas GSI is associated with increased and decreased pNG expression in just three 2° clusters (linear mixed-effects regression assuming beta-binomially distributed gene scores, asterisks indicate significance at α=0.05 after adjusting for a 5% False Discovery Rate). (B) Largely distinct gene-defined subpopulations within clusters show building-, quivering-, and gonadal-associated gene expression. (C) The most significant building-associated pNG expression is observed in 8_Glut (gray lines link paired building/control males), n=38 biologically independent animals (n=19 building, n=19 control). In box plots, the center line indicates the median, the bounds of the box indicate the upper and lower quartiles, and whiskers indicate 1.5x interquartile range. Asterisk indicates significance at α=0.05 after adjusting for a 5% False Discovery Rate. Source data are provided as a Source Data file. (D) Gene-defined populations that exhibit building-associated pNG expression are disproportionately defined by genes encoding receptors and ligands (Fisher’s Exact Test, two-sided). (E) The strongest building-associated pNG expression tends to occur in populations defined by neuromodulatory receptors (Fast Gene Set Enrichment Analysis that uses adaptive multi-level split Monte-Carlo approach for p-value estimation, one-sided, Benjamini-Hochberg adjustment for multiple comparisons), and (F) most strongly in populations that express estrogen receptors (Fast Gene Set Enrichment Analysis that uses adaptive multi-level split Monte-Carlo approach for p-value estimation, one-sided, Benjamini-Hochberg adjustment for multiple comparisons). (G) RG exhibit building-associated *cyp19a1* expression (gray lines link paired building/control males), n=38 biologically independent animals (n=19 building, n=19 control). In box plots, the center line indicates the median, the bounds of the box indicate the upper and lower quartiles, and whiskers indicate 1.5x interquartile range. Asterisk indicates significance at α=0.05 after adjusting for a 5% False Discovery Rate. Source data are provided as a Source Data file. Fish artwork in panel A is reprinted from iScience, Vol 23 / Issue 10, Lijiang Long, Zachary V. Johnson, Junyu Li, Tucker J. Lancaster, Vineeth Aljapur, Jeffrey T. Streelman, Patrick T. McGrath, Automatic Classification of Cichlid Behaviors Using 3D Convolutional Residual Networks, 2020, with permission from Elsevier.


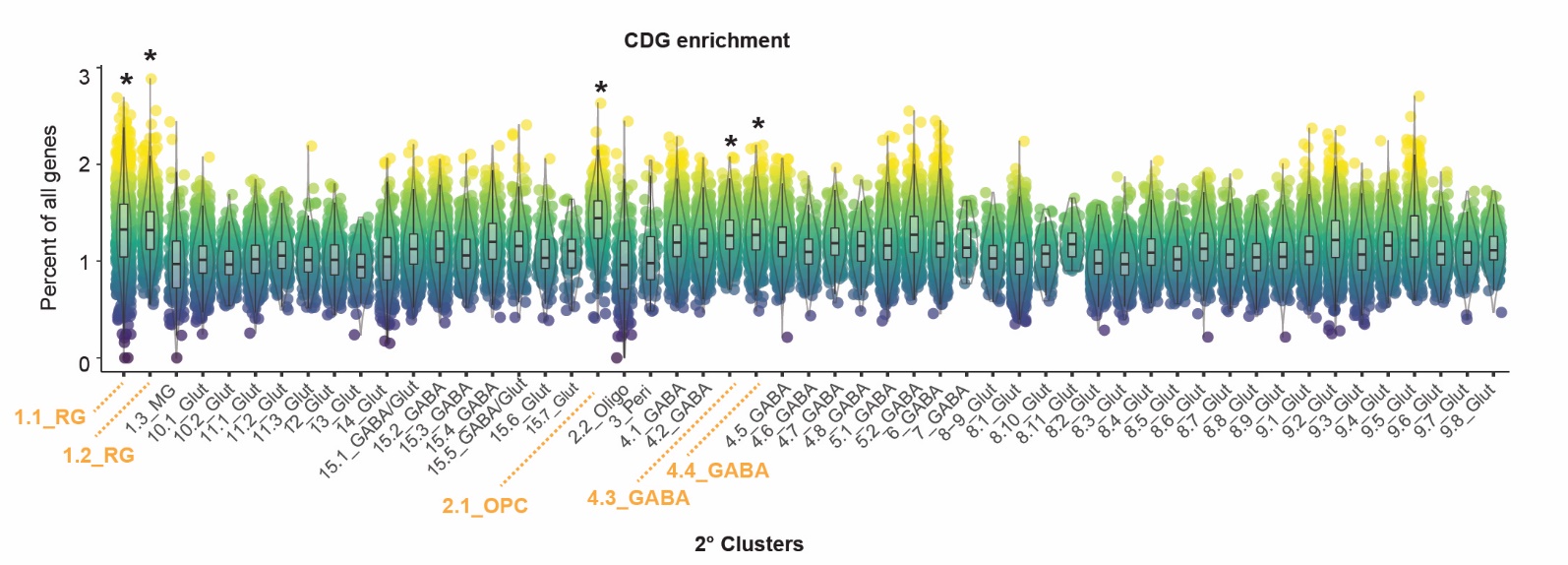


**Figure S8. CDG enrichment across clusters.** CDGs (n=165) are most strongly enriched in non-neuronal populations (y-axis shows the percentage of genes expressed in each nucleus that were CDGs), n=33,674 individual nuclei distributed across 53 2° clusters. In all box plots, the center line indicates the median, the bounds of the box indicate the upper and lower quartiles, and whiskers indicate 1.5x interquartile range. Asterisks indicate effects that were significant after adjustment for 5% False Discovery Rate and additionally as measured by a second permutation test. Source data are provided as a Source Data file.


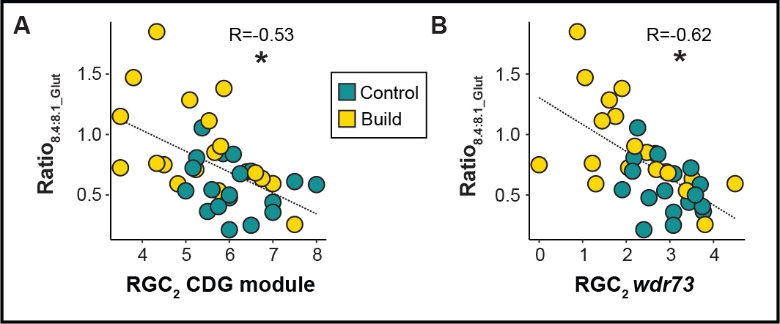


**Figure S9. CDG module and wdr73 expression in RG2 are associated with neuronal rebalancing.** (A) CDG module and (B) *wdr73* expression in RG_2_ are negatively correlated with building-associated neuronal rebalancing between 8.4_Glut and 8.1_Glut (Pearson’s R, two-sided), a pattern that was observed within building males but not within controls. Asterisks indicate effects that were significant at α=0.05.

­
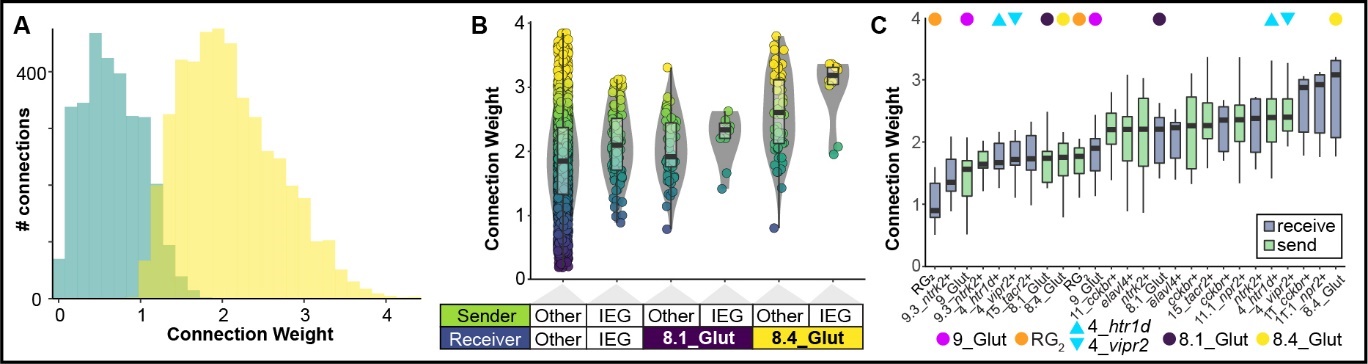


**Figure S10. Strong predicted connections from build-IEG+ senders to 8.4_Glut.** (A) Cell-cell communication connection weights of randomly permuted populations (turquoise) are weaker than connection weights among clusters and build-IEG+ populations. (B) Connection weights among categories of interest; connection weights among build-IEG+ populations (n=100 sender-to-receiver connection weights between build-IEG+ populations) are greater than among other neuronal populations (n=3,136 sender-to-receiver connection weights between other neuronal populations, excluding 8.1_Glut and 8.4_Glut); connection weights between build-IEG+ populations (senders) and 8.4_Glut (receiver, n=10 sender-to-receiver connection weights) are greater than connection weights between other neuronal populations (senders) and 8.4_Glut (receiver, n=56 sender-to-receiver connection weights), and also compared to connection weights between build-IEG+ populations (senders) and 8.1_Glut (receiver, n=10 sender-to-receiver connection weights), and compared to other neuronal populations (senders) and 8.1_Glut (receiver, n=56 sender-to-receiver connection weights). Source data are provided as a Source Data file. (C) Connection weights with 8.4_Glut as a receiver were greater than sending and receiving connection weights for other populations of interest; n=13 sender-to-receiver connection weights in each box plot, representing weights between a given population of interest and build-IEG+ populations, 8.1_Glut, 8.4_Glut, and RG2. In all box plots, the center line indicates the median, the bounds of the box indicate the upper and lower quartiles, and whiskers indicate 1.5x interquartile range. Source data are provided as a Source Data file.


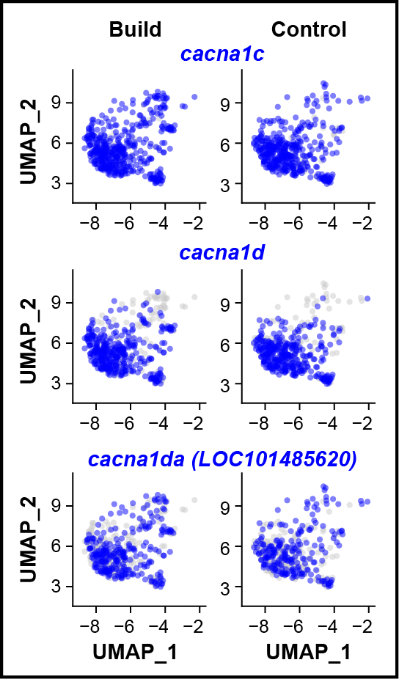


**Figure S11. Gene expression signatures of estrogen sensitivity in build-IEG+ population 4_GABA *vipr2+*.** 4_GABA *vipr2*+ shows strong expression of several genes (blue) encoding estrogen-sensitive L-type Ca^2+^ channels.


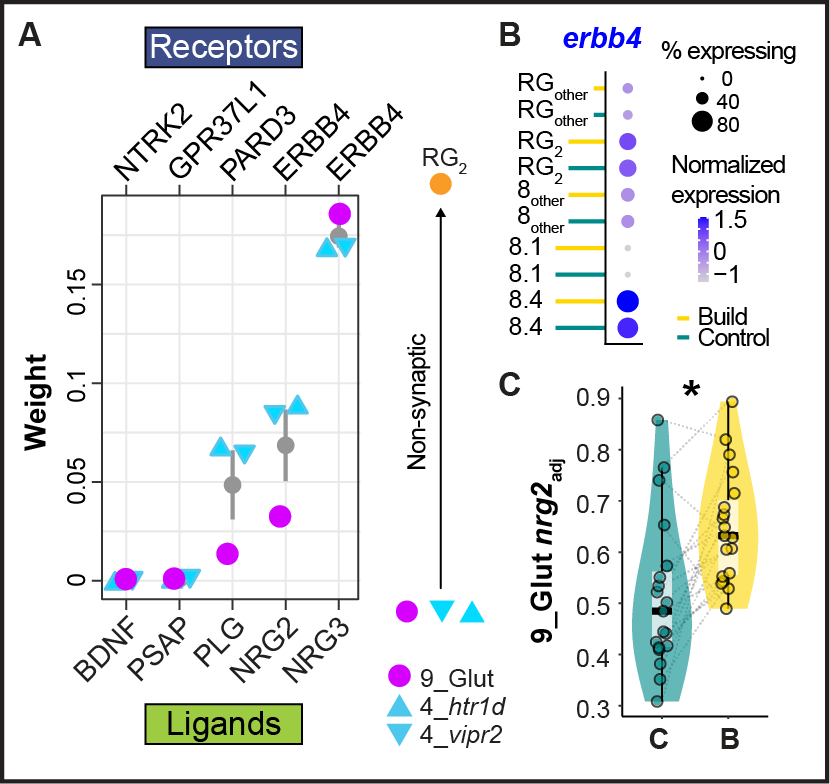


**Figure S12. A candidate molecular spillover pathway linking building-associated neuronal excitation to radial glia and neuronal differentiation.** (A) NRG3-ERBB4 and NRG2-ERBB4 are the strongest cell-cell molecular signaling pathways identified between 9_Glut, 4_GABA *htr1d*+, and 4_GABA *vipr2*+ (senders) and RG_2_ (receiver); n=3 ligand-receptor weights represented in each column, color-coded by cell population; data are presented as mean values +/- SEM. Source data are provided as a Source Data file. (B) *nrg2* shows building-associated upregulation in 9_Glut. (C) *erbb4* shows preferential expression in both RG_2_ and 8.4_Glut, n=38 biologically independent animals (n=19 building, n=19 control). In box plots, the center line indicates the median, the bounds of the box indicate the upper and lower quartiles, and whiskers indicate 1.5x interquartile range. Gray lines link paired building/control males. Asterisk indicates significance at α=0.05 after adjustment for 5% False Discovery Rate. Source data are provided as a Source Data file.


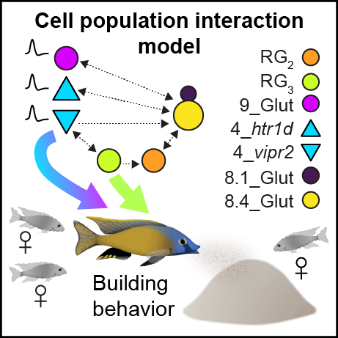


**Figure S13. A testable circuit model for neural regulation of bower-building behavior.** A neuron-glia interaction model for building behavior based on results from IEG, comparative genomics, spatial transcriptomics, mediation, and cell-cell communication analyses. Black arrows represent hypothesized interactions among populations and large colored arrows represent hypothesized causal regulators of bower building behavior. Fish artwork in panel B is reprinted from iScience, Vol 23 / Issue 10, Lijiang Long, Zachary V. Johnson, Junyu Li, Tucker J. Lancaster, Vineeth Aljapur, Jeffrey T. Streelman, Patrick T. McGrath, Automatic Classification of Cichlid Behaviors Using 3D Convolutional Residual Networks, 2020, with permission from Elsevier.

**SUPPLEMENTARY RESULTS**

**Nuclei samples**

A total of 33,674 nuclei (16,975 nuclei from behave pools, 16,669 nuclei from control pools, ranging from 2,053-4,315 nuclei across pools) were mapped to 38 test subjects using genomic DNA. On average, ~800-900 nuclei were sampled from each subject (median=804.5 nuclei, mean=886±46.2 nuclei, range=470-1,568 nuclei per subject). ~1,400 annotated cichlid genes were sampled from each nucleus across subjects (1,422±18.7 genes per nucleus in behaving subjects, 1,322±19.8 genes per nucleus in control subjects). ~18,000 (17,629/32,471 annotated genes; ~54%) genes were detected in all 19 build-control pairs, and ~26,000 (26,119/32,471 annotated genes; ~80%) genes were detected in all ten pools.

**Relationships among building, quivering, and gonadal physiology**

BAI was significantly associated with quivering behavior (Fig. S1; BAI versus log-normalized predicted quivering events, t_36_=4.74, R^2^=0.38, p=3.35x10^-5^) and trended toward a positive association with relative gonadal mass (Fig. S1; BAI versus gonadal somatic index, GSI, t_36_=1.89, R^2^=0.09, p=0.066). We performed mediation and moderation analyses to investigate directional relationships among building, quivering, and gonadal physiology. Quivering and GSI were not identified as possible mediators of differences in building activity levels between control and building males (indirect effect of quivering on the relationship between condition and bower activity index: 0.17±0.34, p=0.564; GSI: 0.002±0.087, p=0.99). Moderation analysis also showed that quivering and GSI were not predicted moderators of building activity levels between groups (group*quivering interaction effect on bower activity index: F_1,32_=0.04, p=0.85; group*GSI interaction effect on bower activity index: F_1,32_=0.01, p=0.92). These data support a model in which building, quivering, and gonadal mass are regulated independently of one another.

**Cluster biology**

We observed two overarching gene expression patterns that distinguished clusters. First, genes encoding nTFs and ligands were overrepresented among cluster marker genes compared to genes encoding receptors (29/43 nTF versus 38/108 receptor genes were 1° markers,  Odds Ratio=3.78, 95% Confidence Interval, CI_95_=[1.70,8.75], p=4.90x10^-4^, FET; 38/43 nTF versus 65/108 receptor genes were 2° markers,  Odds Ratio=4.98, CI_95_=[1.76,17.51], p=8.33x10^-4^; 23/35 ligand versus 38/108 receptor genes were 1° markers, Odds Ratio=3.50, CI_95_=[1.48,8.64], p=0.0028; 30/35 ligand versus 65/108 receptor genes were 2° markers,  Odds Ratio=3.94, CI_95_=[1.36,14.02], p=0.0068) but not compared to each another (29/43 nTF versus 23/35 ligand genes were 1° cluster-specific marker genes, Odds Ratio=1.08, CI_95_=[0.38,3.08], p=1; 38/43 nTF versus 30/35 ligand genes were 2° cluster-specific marker genes, Odds Ratio=1.26, CI_95_=[0.26,6.05], p=0.75). Second, several nTFs involved in dorsal-ventral patterning in early neural development exhibited striking polarity in expression across clusters (Fig. 1G). For example, *dlx* genes and *isl1* mark the ventral telencephalon while *emx* genes mark the dorsal telencephalon during the neurula stage (*1*). These data suggest that transcriptional signatures of developmental patterning are present in adult neurons. Together these data may reflect organizing principles whereby transcriptional programs related to neurodevelopment and ligand synthesis are less labile, whereas neuromodulatory receptors are expressed more promiscuously across cell populations.

**Behavior- and gonadal-associated IEG expression**

*Quivering-associated IEG expression*

In contrast to castle-building, quivering behavior was associated with increased IEG expression in 5_GABA (estimated effect of log-normalized quivering events in model including both condition and GSI as additional fixed effects, β_quiver_=0.090; hmp_adj_=0.0066), a cluster distinguished by expression of a large number of genes expressed in the subpallium and olfactory bulb granule cell layer (e.g. *tac1*, *pax6*, *trh*, *drd1*, *drd2*, *th*, *dat*, *vmat*, *cartpt*, *npy*, and *kiss1r*; Supplementary Data 2). Within 5_GABA, the strongest quivering-associated IEG expression was observed in gene-defined subpopulation 5_GABA *vipr2+* (encodes the vasoactive intestinal peptide receptor 2; β_quiver_=0.232, hmp_adj_=0.0090), followed by 5_GABA *nmbr+* (encodes the neuromedin R receptor; β_quiver_=0.183, hmp_adj_=1.39x10^-4^) and 5.2_GABA *etv1+* (encodes the ETS Variant Transcription Factor 1, which contributes to differentiation of dopaminergic neurons in the olfactory bulb, β_quiver_=0.173, hmp_adj_=0.0016).  Notably, a trend towards strong quivering-associated IEG expression was also observed in 5.2_GABA *th+* (β_quiver_=0.166, hmp_adj_=0.038, p≤0.067 for all models) and 5.1_GABA *drd1+* (LOC101477361, β_quiver_=0.246, hmp_adj_=0.035, p≤0.087 for all models), together raising the possibility that dopaminergic circuits in the olfactory bulb granule cell layer play an important role in the neural coordination of quivering. Two additional 2° clusters exhibited quivering-associated IEG expression, including 8.3_Glut (β_quiver_=0.111, hmp_adj_=0.0035), within which quivering-associated IEG expression was strongest in 8.3_Glut *htr4+* (LOC101474761, encodes serotonin receptor 4; β_quiver_=0.207, hmp_adj_=0.0024); and 9.5_Glut (β_quiver_=0.181, hmp_adj_=0.0036), within which quivering-associated IEG expression was strongest in 9.5_Glut *stmn1+* (LOC101477131, encodes stathmin 1, a marker of immature neurons; β_quiver_=0.322, hmp_adj_=9.10x10^-4^). Analysis of gene-defined populations regardless of cluster identity revealed strong quivering-associated IEG expression in *gcgr+* nuclei (encodes glucagon receptor; β_quiver_=0.161, hmp_adj_=0.0054) and in *slc6a3+* nuclei (encodes dopamine transporter 1, a marker of dopamine-synthesizing neurons; β_quiver_=0.135, hmp_adj_=0.0019). The latter result further supports a potential role for dopaminergic signaling in the neural coordination of quivering, consistent with the role of dopamine in regulating male courtship and mating behaviors in *Drosophila* and other vertebrate lineages (*2*–*5*). In our data, *th+* nuclei strongly localized to 5.2_GABA, a cluster enriched for genetic markers of olfactory granule cell neurons. Dopamine has previously been shown to modulate synaptic transmission between granule and mitral cells in the teleost olfactory bulb (*6*), and our results thus raise the possibility that this circuit plays a central role in male quivering, perhaps modulating responses to chemosensory cues emitted by gravid females. Relative to castle-building, quivering was associated with increased IEG expression in a larger number of clusters and gene-defined cell populations, suggesting that quivering may be associated with more widespread neuronal excitation than castle-building.

*Gonadal-associated IEG expression*

Gonadal steroid hormones can increase Ca^2+^ influx, e.g. via estradiol-mediated Ca^2+^ influx via L-type Ca^2+^ channels (*7*), thereby increasing neuronal excitability. Steroid hormones are synthesized in the gonads, and GSI is correlated with circulating steroid hormones in cichlids (*8*, *9*). Because Ca^2+^ influx triggers IEG expression, we hypothesized that GSI may also be associated with cell type-specific IEG expression. Gonadal-associated IEG expression was not observed in any 1° clusters, but was observed in 4.8_GABA (estimated effect of GSI in model including both condition and log-normalized quivering events as additional fixed effects, β_GSI_=0.801, hmp_adj_=0.0129) and in a large suite of gene-defined populations (Supplementary Data 6). Five gene-defined subpopulations exhibited stronger effects than 4.8_GABA, including (by order of effect size) 8_Glut *galr1+* (encodes galanin receptor 1, β_GSI_=1.01, hmp_adj_=0.0023), 5.1_GABA *oprm1+* (LOC101482911, encodes mu opioid receptor, β_GSI_=0.984, hmp_adj_=0.017), 15_GABA/Glut *htr4+* (LOC101474Fig. 236, encodes serotonin receptor 4, β_GSI_=0.950, hmp_adj_=0.019),  9.3_Glut *nmbr+* (encodes neuromedin B receptor, β_GSI_=0.927, hmp_adj_=0.0052), and 8.5_Glut *sstr2+* (LOC101485655, encodes somatostatin receptor 2, β_GSI_=0.873, hmp_adj_=0.010). These neuronal populations may be “primed” by social contexts that trigger increased gonadal mass, initiating a cascade of steroid-hormone synthesis, release, and subsequent steroid hormone-mediated Ca^2+^ influx.

*Patterns of overlapping behavior- and gonadal-associated IEG expression*

Overall, there was little overlap between cell populations displaying building-, quivering-, or GSI-associated IEG expression, with just two populations showing IEG expression in association with multiple categories: *cckbr+* nuclei (building- and gonadal-associated IEG expression) and 9_Glut *htrd4+* nuclei (quivering- and gonadal-associated IEG expression). One speculative hypothesis is that these two populations are primed by gonadal steroid hormones and also regulate courtship (building, quivering) behaviors. Generally, however, the non-overlapping patterns of building-, quivering-, and gonadal-associated IEG expression across cell populations are consistent with 1) a large degree of molecular and cellular specificity underlying building and quivering, despite both behaviors being expressed in mating contexts, and similarly 2) a large degree of molecular and cellular specificity underlying the neural coordination and/or consequences of male reproductive behaviors (building, quviering) versus male gonadal steroid hormone signaling, despite their co-occurrence over the course of mating. An important caveat is that correlations among these variables may have impeded detection of additional overlapping IEG signals.

*Analysis of building-IEG+ populations as possible mediators of building behavior*

We performed regularized multiple mediation analyses to identify candidate build-IEG+ populations that may mediate building behavior (as measured by BAI). This analysis identified a subset of 5/10 build-IEG+ populations as possible mediators of building behavior, including 9_Glut, 4_GABA *htr1d*+, 4_GABA *vipr2*+, *ntrk2+*, and 11.1_Glut *npr2+*, together explaining a significance amount of variance building activity levels between groups (indirect effect mean=0.41, 95% CI=[0.05,0.72]). These results elevate five build-IEG+ populations as top candidate regulators of behavior.

**Behavior- and gonadal-associated gene expression**

*Build-DEGs, quiver-DEGs, and gonad-DEGs are overrepresented in distinct clusters*

Build-DEGs were overrepresented in three excitatory neuronal clusters (8_Glut, 109/358 1° build-DEG effects, Χ^2^(1, *N*=358)=54.60, q=1.78x10^-12^, Chi-Square Test, CST; 9_Glut, 87/358 effects, Χ^2^(1, *N*=358)=32.27, q=8.06x10^-8^; and 10_Glut, 65/358 effects, Χ^2^(1, *N*=358)=14.19, q=3.31x10^-4^); quiver-DEGs were overrepresented in two neuronal clusters (15_GABA/Glut, 59/224 1° quiver-DEG effects, Χ^2^(1, *N*=224)=24.10, q=3.87x10^-6^, CST; 11_Glut, 37/224 effects, Χ^2^(1, *N*=224)=6.16, q=0.047); and gonad-DEGs were overrepresented in a single inhibitory neuronal cluster (5_GABA, 77/368 1° gonad-DEG effects, Χ^2^(1, *N*=368)=7.11, q=0.018). These data indicate that non-overlapping cell populations were major drivers of building-associated, quivering-associated, and gonadal-associated gene expression. Analysis in 2° clusters aligned closely with these patterns and revealed additional candidate cell populations that were not identified by analyses of 1° clusters. build-DEGs were again overrepresented exclusively in excitatory populations (8.3_Glut, 47/593 2° build-DEG effects, Χ^2^(1, *N*=593)=13.96, q=1.64x10^-3^, CST; 8.9_Glut, 57/593 effects, Χ^2^(1, *N*=593)=22.00, q=3.18x10^-5^; 9.7_Glut, 57/593 effects, Χ^2^(1, *N*=593)=22.00, q=3.18x10^-5^; 10.1_Glut, 66/593 effects, Χ^2^(1, *N*=593)=29.94, q=1.56x10^-6^). Likewise, quiver-DEGs were overrepresented in 15.2_GABA (43/583 2° quiver-DEG effects, Χ^2^(1, *N*=583)=11.35, q=0.013, CST) and were elevated, but not significantly, in 11.1_Glut (29/583 effects, Χ^2^(1, *N*=583)=2.93, q=0.18). quiver-DEGs were also overrepresented in 8.6_Glut (57/583 2° quiver-DEG effects, Χ^2^(1, *N*=583)=22.43, q=7.62x10^-5^, CST). gonad-DEGs were abundant, but not overrepresented, in 5.1_GABA (46/1,145 2° gonad-DEG effects, Χ^2^(1, *N*=583)=1.99, q=0.35, CST), but were overrepresented in 5.1_GABA compared to 5.2_GABA (46/1,145 versus 25/1,145 2° gonad-DEG effects, Odds Ratio=1.87, CI_95_=[1.12,3.21], p=0.015, Fisher’s Exact Test). gonad-DEGs were also overrepresented in two 2° populations within 8_Glut (8.3_Glut, 105/1,145 2° gonad-DEG effects, Χ^2^(1, *N*=1,145)=39.26, q=1.30x10^-8^, CST; 8.4_Glut, 93/1,145 effects, Χ^2^(1, *N*=1,145)=29.58, q=9.38x10^-7^). Notably, both build-DEGs and gonad-DEGs were overrepresented in 8.3_Glut, raising the possibility that this population may integrate gonadal hormonal signals into circuits that regulate building. Taken together, these results show that a disproportionate amount of behavior- and gonadal-associated gene expression is driven by relatively small subsets of neuronal populations, and highlight four 2° excitatory neuronal populations as particularly strong candidates for functional involvement in castle-building.

*Behavior-associated DEGs are differentially enriched for genes related to sodium influx and regulation of neurotransmitter levels, trafficking, and secretion*

Despite the strong overlap of GO terms enriched among upregulated build-DEGs, quiver-DEGs, and gonad-DEGs, differential enrichment testing revealed that genes exhibiting behavior-associated upregulation (upregulated build-DEGs and quiver-DEGs) were differentially enriched for five GO Biological Processes and two GO Cellular Components compared to genes exhibiting gonadal-associated upregulation (upregulated gonad-DEGs). All seven categories were related to either sodium influx or neurotransmitter synthesis, release, and reuptake (“neurotransmitter secretion,” 219 annotated genes in term, 38/530 queried build-DEGs, 31/487 queried quiver-DEGs, 20/544 queried gonad-DEGs, hmp_adj_=0.034; “signal release from synapse,” 219 annotated genes in term, 38/530 queried build-DEGs, 31/487 queried quiver-DEGs, 20/544 queried gonad-DEGs, hmp_adj_=0.034; “exocytic vesicle,” 283 annotated genes in term, 35/535 queried build-DEGs, 29/491 queried quiver-DEGs, 18/547 queried gonad-DEGs, hmp_adj_=0.037; “regulation of neurotransmitter levels,” 285 annotated genes in term, 40/530 queried build-DEGs, 32/487 queried quiver-DEGs, 21/544 queried gonad-DEGs, hmp_adj_=0.027; “neurotransmitter transport,” 275 annotated genes in term, 40/530 queried build-DEGs, 36/487 queried quiver-DEGs, 22/544 queried gonad-DEGs, hmp_adj_=0.027; “sodium ion transport,” 253 annotated genes in term, 21/530 queried build-DEGs, 24/487 queried quiver-DEGs, 10/544 queried gonad-DEGs, hmp_adj_=0.017; “sodium channel complex,” 28 annotated genes in term, 7/535 queried build-DEGs, 8/491 queried quiver-DEGs, 1/547 queried gonad-DEGs, hmp_adj_=0.032). Compared to upregulated gonad-DEGs, upregulated behavior DEGs were also differentially enriched for a suite of protein interactions pathways (n=8, SYK, ACIN1, ERH, BAZ1A, NPEPP5, PARD3B, CKB, and SPTBN4; hmp_adj_<0.05 for all). Taken together, these data suggest that genes upregulated in association with behavior more strongly reflect sodium influx, neurotransmission, and specific molecular pathways than genes upregulated in association with greater GSI.

*Behavior- and gonadal-associated DEGs are enriched for Estrogen Response Elements*

Estrogen and its target receptors (estrogen receptor α, ERα, encoded by *esr1*; and estrogen receptor β, ERβ, encoded by *esr2*) play a major role in vertebrate social behaviors. As a steroid hormone, estrogen can cross the plasma membrane and bind to intracellular ERα or ERβ, forming a complex that translocates into the nucleus and directly modulates gene expression by acting as a transcription factor at estrogen response elements. To investigate the potential role of estrogen signaling in behavior- and gonadal-associated gene expression, we tested if genes containing or immediately proximate to estrogen response elements were overrepresented among build-DEGs, quiver-DEGs, and gonad-DEGs. Remarkably, we found that genes containing estrogen response elements (150/32,471, ~0.46% of all annotated genes; Fig. 4E, gray) were strongly and independently overrepresented among build-DEGs (22/656, ~3.4% build-DEGs; Fig. 4E, yellow), quiver-DEGs (10/609 ~1.6% quiver-DEGs; Fig. 4E, purple), and gonad-DEGs (27/1,028, ~2.6% gonad-DEGs; Fig. 4E, green), consistent with a role for estrogen signaling in regulating building-, quivering-, and gonadal-associated gene expression. 18 DEGs containing estrogen response elements (ERE-DEGs) were represented across multiple categories (build-DEGs, quiver-DEGs, gonad-DEGs) but in largely distinct subsets of 1° and 2° clusters. For example, three ERE-DEGs, *cadm2* (encodes Cell Adhesion Molecule 2), *ptprd* (encodes protein Tyrosine Phosphatase Receptor Type D), and *oxr1* (encodes Oxidation Resistance Type 1, a gene associated with neurodegenerative diseases) (*10*), each exhibited building-, quivering-, and GSI-associated expression but in distinct clusters. These results are consistent with overlapping molecular systems regulating common transcriptional programs in a cell type-specific manner during building, quivering, and shifts in hormonal states. Notably, build-ERE-DEGs were most strongly represented in 8_Glut (4/11 1° build-ERE-DEG effects) and 10.1_Glut (5/22 2° build-ERE-DEG effects), supporting estrogen signaling in these populations as a candidate mechanism underlying castle-building behavior.

**Relationships among rebalancing, neuronal excitation, and behavior**

We hypothesized that rebalancing, or changes in the relative proportions of 8.4_Glut and 8.1_Glut, may be causally related to building and/or to building-associated neuronal excitation. Although 8.1_Glut proportion and the relative difference in proportions of 8.4_Glut and 8.1_Glut were identified as candidate mediators of building, their combined indirect effect on building activity levels was not significant (indirect effect mean=0.15, CI_95_=[-0.04,0.33]). We found stronger evidence for rebalancing as a possible mediator of build-IEG+ excitation. Rebalancing was identified as a possible mediator of IEG score in two build-IEG+ populations: 9_Glut (indirect effect mean=0.62, CI_95_=[0.05,1.31]) and 4_GABA *htr1d+* (indirect effect mean=0.82, CI_95_=[0.54,1.45]), both of which were previously identified as potential mediators of building behavior (see above results under “Analysis of building-IEG+ populations as possible mediators of building behavior”). A follow-up analysis additionally supported rebalancing as a possible moderator of 9_Glut excitation (interaction effects condition x 8.4_Glut proportion on 9_Glut IEG score, F_1,30_=5.78, p=0.022; condition x 8.1_Glut proportion, F_1,30_=3.33, p=0.078), but not of 4_GABA *htr1d+* excitation. This pattern was characterized by a positive relationship between 8.4_Glut proportion and 9_Glut IEG score among building (R=0.39) but not control (R=-0.23) males. We also found evidence for mediation of rebalancing by build-IEG+ excitation. Four build-IEG+ populations (9_Glut, 4_GABA *htr1d+*, 4_GABA *vipr2+*, *elavl4+*) were identified as possible mediators of changes in both 8.1_Glut proportion (combined indirect effect mean=0.90, CI_95_=[0.77,1.23]), 8.4_Glut proportion (combined indirect effect mean=0.67, CI_95_=[0.18,1.25]), and the difference in their relative proportions (combined indirect effect mean=0.77, CI_95_=[0.46,1.03]). The strongest mediation effects on changes in the relative balance between 8.1_Glut and 8.4_Glut were led by 4_GABA *htr1d+* (indirect effect mean=-0.31, CI_95_=[-0.62,-0.10]) and 9_Glut (indirect effect mean=-0.14, CI_95_=[-0.35,0.03]). Notably, both mediation relationships were stronger in the reverse direction (i.e. rebalancing as a mediator of 9_Glut and 4_GABA *htr1d+* IEG score). Together these results support a model in which rebalancing modulates excitation of build-IEG+ neuronal populations that in turn regulate building behavior.

**Behavior- and gonadal-associated pNG expression**

*Castle-building is associated with widespread and cell type-specific expression of genes that positively regulate neurogenesis*

Most (6/7) clusters enriched for build-DEGs did not exhibit building-associated IEG expression, suggesting that a large proportion of building-associated gene expression was most likely explained by factors other than building-associated neuronal activation. Based on building-associated changes in cell proportions and enrichment of neurogenesis-related GO categories among build-DEGs, we hypothesized that neurogenesis and/or processes related to neuronal morphogenesis (e.g. axon growth, dendritic branching) may play a central role in castle-building and partially explain patterns of building-associated gene expression. To investigate this, we identified 87 genes with the GO annotation “positive regulation of neurogenesis” in both zebrafish and mice (“proneurogenic” genes, pNGs), and analyzed their behavior- and gonadal-associated expression with the same approach used for analysis of IEG expression. However, because the typical course of neurogenesis proceeds over timescales exceeding two hours (*11*), we required effects to be significantly associated with condition (building versus not) but not necessarily with BAI (the amount of building activity in the ~100 minute period preceding euthanization), and we instead noted instances in which both criteria were met. Building was associated with increased pNG expression in six 1° neuronal clusters (8_Glut, β_build_=0.065, hmp_adj_=1.23x10^-17^; 9_Glut, β_build_=0.085, hmp_adj_=6.87x10^-14^; 10_Glut, β_build_=0.075, hmp_adj_=2.11x10^-4^; 11_Glut, β_build_=0.062, hmp_adj_=1.48x10^-4^; 4_GABA, β_build_=0.053, hmp_adj_=8.32x10^-9^; and 15_GABA/Glut, β_build_=0.048; hmp_adj_=3.65x10^-7^), and in ten aligned 2° neuronal clusters (8.2_Glut, β_build_=0.076, hmp_adj_=1.50x10^-4^; 8.4_Glut, β_build_=0.076, hmp_adj_=0.028; 8.9_Glut, β_build_=0.123, hmp_adj_=0.0026; 8-9_Glut, β_build_=0.125, hmp_adj_=0.020; 9.1_Glut, β_build_=0.125, hmp_adj_=2.22x10^-7^; 9.6_Glut, β_build_=0.120, hmp_adj_=0.0012; 10.1_Glut, β_build_=0.103, hmp_adj_=5.83x10^-6^; 11.1_Glut, β_build_=0.065, hmp_adj_=0.0039; 4.4_GABA, β_build_=0.101, hmp_adj_=7.28x10^-4^; and 4.5_GABA, β_build_=0.114; hmp_adj_=0.0043 for all). In contrast to building, GSI was associated with increased pNG expression in 10.2_Glut (β_GSI_=0.170, hmp_adj_=0.010) and decreased pNG expression in 4.8_GABA (β_GSI_=-0.343, hmp_adj_=0.0048), whereas quivering was not associated with pNG expression in any 1° or 2° cluster.

*Building-associated pNG expression in clusters is driven by small gene-defined subpopulations*

Notably, building-associated pNG expression was stronger (greater effect estimates, β_build_) in 2° clusters than in corresponding parent 1° clusters. Similarly, a large number of subpopulations defined by specific genes of interest exhibited stronger building-associated pNG expression than their embodying “parent” clusters as a whole. For example, within 15_GABA/Glut, building-associated increases in pNG expression were >3x greater in subpopulations defined by expression of *adra2b* (223/3,551, ~6% of all nuclei in 15_GABA/Glut; β_build_=0.188, hmp_adj_=0.0021) and *esr2* (161/3,551, ~5% of all nuclei in 15_GABA/Glut; β_build_=0.154, hmp_adj_=0.040) compared to 15_GABA/Glut as a whole (β_build_=0.048 within 15_GABA/Glut as a whole). The same general pattern was true of gene-defined subpopulations within 2° clusters that exhibited building-associated pNG expression. The most extreme cases included 8.2_Glut *drd4+* nuclei (139/1,055, ~13% of nuclei in 8.2_Glut; hmp_adj_=6.09x10^-5^; β_build_=0.219 in 8.2_Glut *drd4+* nuclei versus β_build_=0.076 in 8.2_Glut), 8.4_Glut *htr4+* nuclei (362/870, ~42% of nuclei in 8.4_Glut; hmp_adj_=0.018; β_build_=0.126 in 8.4_Glut *htr4*+ nuclei versus β_build_=0.076 in 8.4_Glut), 9.1_Glut *sstr5+* nuclei (191/1,273, ~15% of nuclei in 9.1_Glut; hmp_adj_=6.60x10^-4^; β_build_=0.250 in 9.1_Glut *sstr5*+ nuclei versus β_build_=0.133 in 9.1_Glut), 9.6_Glut *htr4+* nuclei (178/635, ~28% of nuclei in 9.6_Glut; hmp_adj_=6.06x10^-4^; β_build_=0.213 in 9.6_Glut *htr4*+ nuclei versus β_build_=0.120 in 9.6_Glut), 10.1_Glut *ntrk2+* nuclei (326/1,475, ~22% of nuclei in 10.1_Glut; hmp_adj_=5.78x10^-5^; β_build_=0.227 in 10.1_Glut *ntrk2+* nuclei versus β_build_=0.103 in 10.1_Glut), 11.1_Glut *ntrk2+* nuclei (259/972, ~27% of nuclei in 11.1_Glut; hmp_adj_=0.00626; β_build_=0.134 in 11.1_Glut *ntrk2+* nuclei versus β_build_=0.065 in 11.1_Glut), and 4.5_GABA *bcl11b+* nuclei (385/619, ~62% of nuclei in 4.5_GABA; hmp_adj_=1.24x10^-4^; β_build_=0.191 in 4.5_GABA *bcl11b+* nuclei versus β_build_=0.114 in 4.5_GABA). These results support the idea that relatively small subpopulations embedded within 1° and 2° clusters drive building-associated pNG expression in the telencephalon.

*Populations exhibiting strong pNG expression disproportionately express estrogen receptors*

Populations that exhibited building-associated pNG expression were disproportionately defined by genes associated with neuromodulatory signaling (ligands or receptors) compared to ndTFs (20/35 ligand genes versus 59/108 receptor genes versus 12/43 nTF genes; p=0.0065, FET). When compared directly, populations defined by nTFs were less likely to exhibit building-associated pNG expression compared to populations defined by neuromodulatory receptors (Odds Ratio=0.30, CI_95_=[0.10,0.82], p=0.012, FET) or ligands (Odds Ratio=0.32, CI_95_=[0.14,0.73], p=0.0037). Ranked enrichment testing further showed that populations defined by neuromodulatory receptor genes, but not populations defined by ligands or nTF genes, were overrepresented among populations showing the strongest building-associated increases in pNG expression (as measured by effect size; receptors, Normalized Enrichment Score, NES=1.64, q=0.011; ligands, NES=1.41, q=0.13; nTFs, NES=0.52, q=0.97). To investigate whether specific molecular signaling systems may have especially strong relationships with building-associated neurogenesis, we performed a ranked enrichment test across receptor systems that were represented by at least three unique genes (i.e. serotonin, n=9; adrenergic, n=6; estrogen, n=5; calcitonin, n=4; dopamine, n=4; acetylcholine, n=3; adenosine, n=3; histamine, n=3; natriuretic peptide, n=3; opioid, n=3; relaxin, n=3; somatostatin, n=3; and tachykinin, n=3). This analysis revealed that populations defined by ERs were overrepresented among those exhibiting the strongest building-associated increases in pNG expression (NES=2.00, q=0.034), suggesting a link between estrogen signaling and building-associated neurogenesis.

*Building-associated pNG expression is strongest in nuclei expressed pro-melanin concentrating hormone*

We also investigated building-associated pNG expression in gene-defined populations regardless of cluster identity. Nine gene-defined populations exhibited stronger building-associated pNG expression than any 1° or 2° cluster, including those defined by expression of *pmch* (n=189 nuclei, β_build_=0.340, hmp_adj_=3.76x10^-6^), *ghrhr* (LOC101475240; n=580 nuclei, β_build_=0.239, hmp_adj_=3.33x10^-6^), *th* (n=390 nuclei, β_build_=0.197, hmp_adj_=3.35x10^-4^), *adra2c* (LOC101479598, n=289 nuclei, β_build_=0.157, hmp_adj_=7.75x10^-7^), *crhbp* (LOC101487439, n=698 nuclei, β_build_=0.151, hmp_adj_=4.18x10^-4^), *galr1* (LOC101469593, n=527 nuclei, β_build_=0.147, hmp_adj_=0.00197), *tac1* (n=329 nuclei, β_build_=0.138, hmp_adj_=0.0375), *esr1* (LOC101479381, n=612 nuclei, β_build_=0.134, hmp_adj_=0.00150), and *cd82* (LOC101480351, n=725 nuclei, β_build_=0.127, hmp_adj_=0.00315). pNG expression was additionally associated with BAI in *pmch+* (β_BAI_=0.101, hmp_adj_=3.76x10^-6^) and *ghrhr+* (LOC101475240; β_BAI_=0.051, hmp_adj_=0.00261) nuclei, suggesting a strong relationship between recent behavior and pNG expression in these populations. Notably, *pmch+* nuclei also exhibited the greatest building-associated pNG expression of any population analyzed (i.e. all 1° clusters, 2° clusters, gene-defined populations within clusters, and gene-defined populations regardless of cluster), highlighting this population as an especially strong candidate for functional involvement in castle-building behavior. A single population, defined by expression of *emx2* (encodes Empty Spiracles Homeobox 2), exhibited behavior-associated decreases in pNG expression (β_build_=-0.26, hmp_adj_=5.97x10^-6^).

**Directional relationships among RG biology, neuronal rebalancing, build-IEG+ populations, and building behavior**

We hypothesized that changes in RG biology may be causally related to 8.1_Glut and 8.4_Glut rebalancing, building-associated neuronal excitation, and/or building behavior. We investigated both upstream mediators and downstream effects of RG_2_ quiescent score, RG_3_ *cyp19a1* (the gene encoding aromatase) expression, RG_4_ proportion, and *cyp19a1* expression across all RG. Both RG_3_ *cyp19a1* expression and *cyp19a1* expression across all RG were identified as possible mediators of building behavior (BAI, indirect effect mean=0.33, CI_95_=[0.06,0.61]), with a stronger mediation effect predicted for RG_3_ *cyp19a1* (indirect effect mean=0.27, CI_95_=[0.06,0.51]) than for *cyp19a1* expression across all RG (indirect effect mean=0.09, CI_95_=[-0.10,0.28]). These results are consistent with other strong building-associated estrogen- and ER-related signals in the data, for example ER-expressing populations showing disproportionately strong building-associated pNG expression, and enrichment of estrogen response elements among build-DEGs. Together, these results support a model in which estrogen signaling in the telencephalon regulates castle-building behavior. A simplified model is that building-associated increases in aromatase in RG_3_ lead to increased estrogen synthesis and release, subsequent estrogen binding to cell type-specific estrogen receptors, and positive regulation of building.

Analysis of these RG factors as possible mediators of rebalancing identified RG_2_ quiescent score, RG_3_ *cyp19a1* expression, and RG_4_ proportion, but not *cyp19a1* expression across all RG, as candidate mediators of rebalancing. Together these factors explained a significant amount of variance in group differences in 8.1_Glut proportion (combined indirect effect mean=0.95, CI_95_=[0.80,1.20]), 8.4_Glut proportion (combined indirect effect mean=0.94, CI_95_=[0.64,1.36]), and the difference in relative proportions of 8.1_Glut and 8.4_Glut (combined indirect effect mean=0.93, CI_95_=[0.77,1.23]). These data are consistent with changes in RG subpopulation-specific biology playing a causal role in the rebalancing of 8.1_Glut and 8.4_Glut.

It is thought that hippocampal neurogenesis in mice is regulated by both excitation of local circuits and excitation of outside populations projecting into the hippocampus (*12*). Build-IEG+ populations, particularly 9_Glut and 4_GABA *vipr2+,* were identified as possible mediators of changes in RG biology, particularly RG_3_ *cyp19a1* expression (indirect effect mean=0.61, CI_95_=[0.31,1.05]). IEG score in 4_GABA *vipr2+* had a stronger mediation effect (indirect effect mean=0.25, CI_95_=[0.02,0.46]) than IEG score in 9_Glut (indirect effect mean=0.09, CI_95_=[-0.08,0.23]) on RG_3_ *cyp19a1* expression. Conversely, both RG_3_ *cyp19a1* expression and RG_2_ quiescence were predicted mediators of IEG score in 4_GABA *vipr2+* (combined indirect effect mean=0.91, CI_95_=[0.75,1.25]). Notably, 4_GABA *vipr2+* expresses several estrogen-sensitive L-type Ca^2+^ channels (Fig. S11) (*7*), offering a plausible molecular pathway by which RG_3_ aromatase may regulate 4_GABA *vipr2+* excitability. Together, these data are consistent with a model in which 4_GABA *vipr2+* and RG_3_ interact in a building-associated fashion to jointly promoting downstream rebalancing and building behavior.

**CDG enrichment patterns**

*CDGs are enriched for human brain disease-associated genes*

The 165 CDGs in the 19Mbp region on LG11 were largely conserved in humans (136/165, ~82% had predicted human homologs) and were enriched for GO categories including “proximal/distal pattern formation” 5/37 annotated genes; *HOXA9, HOXA10, HOXA11, IRX1, IRX2*; q=0.012) and “ionotropic glutamate receptor signaling pathway” (4/30 annotated genes; *GRIK3*, *GRIK5*, *GRIN2D*, *ATP1A3*; q=0.032); the butyrophilin gene family (C2-set and V-set domain containing, *MAG, NPHS1, CADM4, KIRREL2, CD22, AGER, NECTIN2, BCAM*; 8/40 annotated genes, q=2.39x10^-9^), and several human brain disease-associated cytobands including 8q23 (6/18 annotated genes; *TRHR,* *OXR1, ZFPM2, MAL2, COL14A1, EBAG9;* q=4.55x10^-9^), 19q13.1 (7/55 annotated genes;  *MAG, NPHS1, FFAR2, APLP1, ZBTB32, CD22, HAMP*; q=8.62x10^-9^), 1p34.3 (7/66 annotated genes;  *GRIK3, MAP7D1, EVA1B, UTP11, GNL2, RSPO1, THRAP3*; q=2.05x10^-7^), and 19q13.2 (9/169 annotated genes; *SPHK2, GRIK5, XRCC1, LIPE, CEACAM21, TMEM145, NECTIN2, KCNN4, BCAM*; q=4.83x10^-7^). Notably, CDGs also included homologs of many human brain disease-associated genes, including 23 genes associated with Parkinson’s Disease, as well as *KIRREL2* (associated with prolonged epileptic seizures) and *PLEC* (associated with limb-girdle muscular dystrophy). These data suggest that castle-building behavior has evolved through variation in conserved and disease-associated genes.

*Specific neuronal and non-neuronal cell populations are enriched for castle-divergent genes*

CDGs (n=165) were enriched in two primary clusters, 1_RG (Cohen’s d=0.63, CI_95_=[0.59,0.68], p<2.2x10^-16^; proportion of permutations with greater effect, p_perm_=0.0006) and 2_OPC/Oligo (Cohen’s d=0.58, CI_95_=[0.50,0.66], p<2.2x10^-16^; p_perm_=0.0095). CDGs were also enriched in five secondary clusters, including neuronal 4.3_GABA (Cohen’s d=0.53, CI_95_=[0.46,0.61], p<2.2x10^-16^; p_perm_=0.0077) and 4.4_GABA (Cohen’s d=0.54, CI_95_=[0.47,0.62], p<2.2x10^-16^; p_perm_=0.023) as well as non-neuronal 1.1_RG (Cohen’s d=0.72, CI_95_=[0.66,0.78], p<2.2x10^-16^; p_perm_=0.001), 1.2_RG (Cohen’s d=0.74, CI_95_=[0.66,0.81], p<2.2x10^-16^; p_perm_=0.0006), and 2.1_OPC (Cohen’s d=1.10, CI_95_=[1.00,1.19], p<2.2x10^-16^; p_perm_=0.002). These data reveal candidate neuronal, RG, and OPC cell populations that may have undergone transcriptional specializations over the course of castle-building evolution.

*CDG enrichment in clusters and gene-defined populations*

The strongest CDG enrichment effect of any gene-defined subpopulation was observed within 2.1_OPC *chrm4+* nuclei (114/431, ~26% 2.1_OPC nuclei, Cohen’s d=1.32, CI_95_=[1.14,1.51], p_perm_=0.0003), but this effect was only modestly (1.2x) stronger compared to the enrichment effect observed in 2.1_OPC as a whole (Cohen’s d=1.10, CI_95_=[1.10,1.19], p_perm_=0.002). Similarly, the strongest effect observed among gene-defined RG subpopulations was 1.1_RG *fabp7+* (197/1,165, ~17% 1.1_RG nuclei, Cohen’s d=0.79, CI_95_=[0.65,0.93], p_perm_=0.0009), and was only modestly (~1.1x) stronger than the enrichment effect observed in 1.1_RG as a whole (Cohen’s d=0.72, CI_95_=[0.66,0.78], p_perm_=0.001).

We also tested for CDG enrichment among gene-defined cell populations regardless of cluster identity. In total, 28 populations (28/203, ~14%) were enriched for CDGs. The genes defining enriched populations were imbalanced across biological categories (p=1.38x10^-4^, FET; excluding “other”), and were disproportionately defined by genes encoding nTFs (n=13/43 genes tested) compared to genes encoding specific neuromodulatory ligands (2/35 genes tested, Odds Ratio=6.99, CI_95_=[1.41,69.00], p=0.0083, FET) or neuromodulatory receptors (6/108 genes tested, Odds Ratio=7.24, CI_95_=[2.33,25.35], p=1.26x10^-4^, FET). These data raise the possibility that brain region-specific and nTF-defined cell lineages may have been targeted in the evolution of castle-building behavior. We observed two additional striking patterns among enriched populations. First, the most strongly enriched populations were defined by a subset of genes expressed almost exclusively in RG. The six most strongly enriched populations were *notch1a+*, *slc1a3+*, *fabp7+*, *notch1b+, s100b+*, and *gfap+* nuclei, all of which are genes expressed with high specificity in RG. *emx2+* and *pdgfrb+* nuclei were also enriched for CDGs and were strongly expressed in RG. Second, many enriched populations were defined by genes associated with RG quiescence/activation and neurogenesis (e.g. *cyp19a1*, *sox2*, *pax6*, *dcn*, *notch3*, *hes1*, *hes5*, *id1*, *ccnd1*, and *etv1*). Notably, two enriched populations were defined by genetic markers of oligodendrocytes (*sox10* and *cd82*), consistent with results in 1° and 2° clusters. Taken together, these data highlight RG and OPCs as candidate cell populations in which transcriptional specializations may have contributed to the evolution of castle-building behavior.

*CDG expression is more strongly associated with signatures of neurogenesis than neuronal activation*

Based on enrichment of CDGs in RG, as well as correlated building-associated signatures of changes in RG functional states and changes in cell type-specific proportions, we reasoned that CDGs may play a role in behavior-associated neurogenesis. We hypothesized that if CDGs are involved in behavior-associated neurogenesis, then CDG expression may be associated with gene expression signatures of neurogenesis. In support of our hypothesis, we found that CDG score was positively associated with pNG score (n=38, R=0.62, t_1,36_=4.71, p=3.58x10^-5^), but not IEG score (n=38, R=0.11, t_1,36_=0.66, p=0.51), across males (pNG score and IEG score were also not associated across males; n=38, R=-0.075, t_1,36_=-0.45, p=0.65). Indeed, CDG score and pNG score were significantly more positively associated compared to CDG score and IEG score (t_1,36_=2.50, p=0.017) and compared to pNG score and IEG score (t_1,36_=3.90, p=4.22x10^-4^). These data support a relationship between CDGs and gene expression programs related to neurogenesis.

**Directional relationships among RG CDG expression, neuronal rebalancing, build-IEG+ populations, and building behavior**

We hypothesized that CDG module expression in RG may be causally related to changes in RG biology, 8.1_Glut and 8.4_Glut rebalancing, building-associated neuronal excitation, and/or building behavior. To investigate this, we conducted mediation analyses in which building-associated CDG module effects in specific RG subclusters (RG_1_ *wdr73* expression, RG_2_ *wdr73* expression, RG_2_ CDG module score, RG_8_ CDG module score) were tested as possible mediators of other changes in RG biology, 8.1_Glut and 8.4_Glut rebalancing, build-IEG+ excitation, and BAI. These analyses supported directional relationships linking CDG expression in RG_2_ and RG_8_ to RG_2_ quiescence, RG_3_ aromatase, and neuronal rebalancing, detailed below.

RG_2_ *wdr73* expression, RG_2_ CDG module score, and RG_8_ CDG module score were predicted mediators of RG_2_ quiescent score (combined indirect effect mean=0.94, CI_95_=[0.66,1.34]) and RG_3_ *cyp19a1* expression (combined indirect effect mean=0.55, CI_95_=[0.28,0.97]). The strongest predicted mediators included RG_2_ *wdr73* expression (indirect effect mean=0.21, CI_95_=[0.04,0.43]) and RG_2_ CDG module score (indirect effect mean=0.15, CI_95_=[0.01,0.33]). Conversely, RG_2_ quiescent score and RG_3_ *cyp19a1* expression were identified as possible mediators of CDG expression in RG, specifically RG_2_ *wdr73* expression (indirect effect mean=0.99, CI_95_=[0.17,1.83]) and RG_2_ CDG module score (indirect effect mean=0.99, CI_95_=[0.76,1.24]). Together, these data are consistent with causal interaction between RG_3_ aromatase and CDG expression in RG_2_, and with causal relationships between RG_2_ CDG expression and RG_2_ quiescence. Notably, RG_2_ preferentially expressed *esr2* at higher levels compared to other RG (41% of RG_2_ nuclei expressed *esr2* versus 26% of other RG nuclei), and activation of ESR2 has previously been shown to reduce stemness of glioma stem cells (*13*). These data are consistent with a model in which building-associated synthesis and release of estrogen via RG_3_ aromatase promotes an exit from quiescence in RG_2_.

RG_2_ *wdr73*, RG_2_ CDG module score, and RG_8_ CDG module score were identified as possible mediators of 8.1_Glut and 8.4_Glut rebalancing, and collectively explained group differences in 8.4_Glut proportions (combined indirect effect mean=0.98, CI_95_=[0.77,1.23]) and the difference in the relative proportions of 8.1_Glut and 8.4_Glut (combined indirect effect mean=0.90, CI_95_=[0.74,1.26]), with the strongest individual mediation effect attributed to RG_2_ *wdr73* expression on the difference in the relative proportions of 8.1_Glut and 8.4_Glut (indirect effect mean=-0.15, CI_95_=[0.32,0.06]). Conversely, rebalancing (8.4_Glut proportion, 8.1_Glut proportion, and the difference in their relative proportions) was identified as a possible mediator CDG expression in RG, specifically of RG_2_ *wdr73* expression (indirect effect mean=0.95, CI_95_=[0.78,1.22]). These patterns are consistent with causal relationships between CDG expression in RG and building-associated hippocampal-like neuronal rebalancing, and with building-associated changes in CDG expression in specific RG subpopulations (RG_2_, RG_8_) shifting patterns of glial quiescence, differentiation, and neurogenesis, ultimately resulting in hippocampal-like cellular reorganization.

We did not find strong evidence for build-IEG+ excitation as a mediator of CDG expression in RG, or vice versa. Similarly, although RG_2_ *wdr73* expression was identified as a possible mediator of building behavior (but not RG_1_ *wdr73* expression, RG_2_ CDG module expression, or RG_8_ CDG module expression), its indirect effect on building activity was not significant (CI_95_=[-0.059, 0.27]). Together these results support CDG expression in RG being most directly and strongly related to RG_3_ *cyp19a1* expression and 8.1_Glut and 8.4_Glut rebalancing.

**Cell-cell communication analysis identifies candidate projections to 8.4_Glut underlying building**

CellChat revealed a trend whereby connection weights among neuronal populations of interest were greater than among other neuronal populations (Fig. S10; p=2.77x10^-7^, Welch two-sample t-test) and among randomly permuted size-matched populations (p_perm_=0 for all). Among neuronal populations of interest, 8.4_Glut (receiver) had the greatest connection weights of any sender or receiver (Fig. S10). We observed a trend whereby build-IEG+ populations had greater sending weights than other neuronal populations to 8.4_Glut (p=0.072, Welch two-sample t-test; but not to 8.1_Glut, p=0.36; Fig. S10), supporting a model whereby populations that fire during building project preferentially to 8.4_Glut.

Approximately 2% (94/4,096) of connection weights exhibited building-associated change, with the vast majority (91/94) reflecting building-associated increases in weight. Unexpectedly, oligodendrocyte precursor cells (2.1_OPC) were more highly represented (as a receiver) among building-associated connection changes than any other population (21/94, Odds Ratio=26.38, CI_95_=[14.14,48.21], p=6.91x10^-20^, FET), and corresponding senders included six build-IEG+ populations, 8.1_Glut, and 8.4_Glut (Supplementary Data 20), possibly reflecting behavior-associated and activity-dependent myelination (*14*). Building was also associated with decreases in connection weight between 1.1_RG (sender), which encompassed RG subpopulations expressing markers of cycling and differentiation, and RG_2_ (receiver), supporting building-associated changes in dynamics between RG_2_ and other RG.

**Mediation analysis supports causal interaction among neural activity, glial function, rebalancing, and building behavior**

Final regularized multiple mediation analyses identified top candidate mediators of building, rebalancing, RG_3_ *cyp19a1* expression, and RG_2_ biology. These analyses tested build-IEG+ scores, rebalancing (8.1_Glut proportion, 8.4_Glut proportion, and difference in relative proportion between 8.1_Glut and 8.4_Glut), RG biology factors (RG_2_ quiescent score, RG_3_ *cyp19a1* expression, RG_4_ proportion, and *cyp19a1* expression across all RG), and RG CDG expression (RG_1_ *wdr73* expression, RG_2_ *wdr73* expression, RG_2_ CDG module score, RG_8_ CDG module score) as possible mediators.

Top candidate mediators of building activity included RG_3_ *cyp19a1*, rebalancing (8.1_Glut proportion, difference in relative proportion between 8.1_Glut and 8.4_Glut), *ntrk2+* IEG score, 9_Glut IEG score, 4_GABA *vipr2+* IEG score, and 11.1_Glut *npr2+* IEG score. Together, these variables explained a significant amount of group differences in BAI (indirect effect mean=0.67, CI_95_=[0.46,1.00]), with the strongest individual mediation effects being RG_3_ *cyp19a1* expression (indirect effect mean=0.23, CI_95_=[0.03,0.38]) and 11.1_Glut *npr2+* IEG score (indirect effect mean=0.23, CI_95_=[0.04,0.33]).

Top candidate mediators of rebalancing included RG_2_ *wdr73*, RG_2_ CDG module score, RG_3_ *cyp19a1* expression, RG_8_ CDG module score, 9_Glut IEG score, and 4_GABA *htr1d+* IEG score. Together, these variables explained a significant amount of group differences in 8.4_Glut proportion (indirect effect mean=0.97, CI_95_=[0.86,1.14]), 8.1_Glut proportion (indirect effect mean=0.99, CI_95_=[0.95,1.05]), and the difference in relative proportions of 8.1_Glut and 8.4_Glut (indirect effect mean=0.91, CI_95_=[0.91,1.09]). The strongest individual candidate mediator was RG_2_ *wdr73* expression on the difference in relative proportion between 8.1_Glut and 8.4_Glut (indirect effect mean=-0.14, CI_95_=[-0.34,-0.011]).

Top candidate mediators of RG_2_ (*wdr73* expression, CDG module score) included 8.4_Glut proportion, the difference in relative proportion between 8.1_Glut and 8.4_Glut, RG_2_ quiescent score, RG_3_ *cyp19a1* expression, and 4_GABA *vipr2+* IEG score. These variables explained a significant amount of group differences in RG_2_ *wdr73* expression (indirect effect mean=1.05, CI_95_=[0.33,1.67]), and RG_2_ CDG module score (indirect effect mean=1.05, CI_95_=[0.42,1.58]), with the strongest individual mediation effect being 8.4_Glut proportion (indirect effect mean=-0.11, CI_95_=[-0.27,0.011]).

Candidate mediators of RG_3_ *cyp19a1* expression included 4_GABA *vipr2+* IEG score, RG_2_ CDG module score, RG_2_ *wdr73* expression, 9.3_Glut *ntrk2+* IEG score, 8.4_Glut proportion, 9_Glut IEG score, RG_8_ CDG module score, and the difference in relative proportions between 8.1_Glut and 8.4_Glut. Collectively these factors explained a large proportion of group differences in *cyp19a1* expression (indirect effect mean=0.81, CI_95_=[0.60,1.18]), with the strongest candidate mediator being 4_GABA *vipr2+* IEG score (indirect effect mean=0.17, CI_95_=[-0.02,0.33]).

Taken together with previous mediation and cell-cell communication analyses, these results are consistent with a circuit model whereby excitation of several build-IEG+ populations regulates building behavior as well as changes in RG_2,_ RG_3_, and hippocampal-like neuronal rebalancing, which in turn regulate build-IEG+ excitation and behavior. One speculative possibility is that collectively, these populations and their interactions may compose a neuron-glia behavioral circuit that is 1) functionally specialized in castle-building lineages via divergence in CDG module expression in RG, 2) positively regulated by estrogen (synthesized by RG_3_ aromatase), and 3) “strengthened” during building via influx/integration of new neurons into the circuit through RG_2_–to-8.4_Glut neuronal differentiation.

**A spillover model for build-IEG+ regulation of rebalancing**

In mammals, one mechanism by which neural activity regulates glial-to-neuronal differentiation is “spillover”, or ligand release, diffusion, and binding to target receptors in the absence of synaptic connections (*15*). Given the weak connection weights between build-IEG+ populations and RG_2_ (Figure S9), we hypothesized that spillover may explain their interaction. Analysis of ligands expressed in 9_Glut, 4_GABA *vipr2+*, and 4_GABA *htr1d+* and target receptors expressed in RG_2_ revealed NRG3-ERBB4 and NRG2-ERBB4 as the strongest ligand-receptor pairs (Fig. S12). The NRG2/NRG3-ERBB4 signaling pathway promotes glial differentiation/migration and hippocampal synapse formation in mammals (*16*, *17*). Notably, *nrg2* showed building-associated upregulation in 9_Glut (Fig. S12), and *erbb4* was preferentially expressed in RG_2_ versus other RG, and in 8.4_Glut versus 8.1_Glut and versus all other 8_Glut nuclei (Fig. S12). These data support a plausible circuit model whereby building behavior is coordinated by activation of multiple neuronal populations with a common projection target, downregulation of an evolutionarily divergent gene module in a subpopulation of glia lining that projection target, and hippocampal-like cellular reorganization (Fig. 9J, Fig. S13).

**SUPPLEMENTARY REFERENCES**

1. J. B. Sylvester, C. A. Rich, C. Yi, J. N. Peres, C. Houart, J. T. Streelman, Competing signals drive telencephalon diversity. *Nat. Commun.* **4**, 1745 (2013).

2. K. Keleman, E. Vrontou, S. Krüttner, J. Y. Yu, A. Kurtovic-Kozaric, B. J. Dickson, Dopamine neurons modulate pheromone responses in Drosophila courtship learning. *Nature*. **489**, 145–149 (2012).

3. S. X. Zhang, D. Rogulja, M. A. Crickmore, Dopaminergic circuitry underlying mating drive. *Neuron*. **91**, 168–181 (2016).

4. M. R. Melis, A. Argiolas, Dopamine and sexual behavior. *Neurosci. Biobehav. Rev.* **19**, 19–38 (1995).

5. M. Fujiwara, A. Chiba, Sexual odor preference and dopamine release in the nucleus accumbens by estrous olfactory cues in sexually naïve and experienced male rats. *Physiol. Behav.* **185**, 95–102 (2018).

6. T. Kawai, H. Abe, Y. Oka, Dopaminergic neuromodulation of synaptic transmission between mitral and granule cells in the teleost olfactory bulb. *J. Neurophysiol.* **107**, 1313–1324 (2012).

7. N. E. Vega-Vela, D. Osorio, M. Avila-Rodriguez, J. Gonzalez, L. M. García-Segura, V. Echeverria, G. E. Barreto, L-type calcium channels modulation by estradiol. *Mol. Neurobiol.* **54**, 4996–5007 (2017).

8. K. P. Maruska, R. D. Fernald, Behavioral and physiological plasticity: rapid changes during social ascent in an African cichlid fish. *Horm. Behav.* **58**, 230–240 (2010).

9. B. A. Alward, A. T. Hilliard, R. A. York, R. D. Fernald, Hormonal regulation of social ascent and temporal patterns of behavior in an African cichlid. *Horm. Behav.* **107**, 83–95 (2019).

10. M. R. Volkert, D. J. Crowley, Preventing neurodegeneration by controlling oxidative stress: The role of OXR1. *Front. Neurosci.* **14**, 611904 (2020).

11. J. B. Aimone, Y. Li, S. W. Lee, G. D. Clemenson, W. Deng, F. H. Gage, Regulation and function of adult neurogenesis: from genes to cognition. *Physiol. Rev.* **94**, 991–1026 (2014).

12. J. Song, R. H. J. Olsen, J. Sun, G.-L. Ming, H. Song, Neuronal circuitry mechanisms regulating adult mammalian neurogenesis. *Cold Spring Harb. Perspect. Biol.* **8**, a018937 (2016).

13. G. R. Sareddy, U. P. Pratap, P. P. Venkata, M. Zhou, S. Alejo, S. Viswanadhapalli, R. R. Tekmal, A. J. Brenner, R. K. Vadlamudi, Activation of estrogen receptor beta signaling reduces stemness of glioma stem cells. *Stem Cells*. **39**, 536–550 (2021).

14. R. Marisca, T. Hoche, E. Agirre, L. J. Hoodless, W. Barkey, F. Auer, G. Castelo-Branco, T. Czopka, Functionally distinct subgroups of oligodendrocyte precursor cells integrate neural activity and execute myelin formation. *Nat. Neurosci.* **23**, 363–374 (2020).

15. R. Pardal, J. López Barneo, Mature neurons modulate neurogenesis through chemical signals acting on neural stem cells. *Dev. Growth Differ.* **58**, 456–462 (2016).

16. W.-J. Zhao, S.-J. Yi, G.-Y. Ou, X.-Y. Qiao, Neuregulin 2 (NRG2) is expressed in gliomas and promotes migration of human glioma cells. *Folia Neuropathol.* **59**, 189–197 (2021).

17. T. Müller, S. Braud, R. Jüttner, B. C. Voigt, K. Paulick, M. E. Sheean, C. Klisch, D. Gueneykaya, F. G. Rathjen, J. R. Geiger, J. F. Poulet, C. Birchmeier, Neuregulin 3 promotes excitatory synapse formation on hippocampal interneurons. *EMBO J.* **37** (2018), doi:10.15252/embj.201798858.
