# Supplementary material for: Cellular profiling of a recently-evolved social behavior in cichlid fishes
Source: Nat Commun. 2023 Aug 14;14:4891. doi: 10.1038/s41467-023-40331-9 (PMC10425353; doi:10.1038/s41467-023-40331-9)
Supplement: Supplementary file 3 — Description of Additional Supplementary Files [file 41467_2023_40331_MOESM3_ESM.pdf]

Description of Additional Supplementary Files

File Name: Supplementary Data 1
Description: Summary data for 38 test subjects.

File Name: Supplementary Data 2
Description: Summary data for marker genes of interest by primary cluster (Sheet 1) and Secondary Cluster (Sheet 2), brain region and cell type-specific expression patterns in teleosts (Sheet 3), and references (Sheet 4).

File Name: Supplementary Data 3
Description: Summary data for primary and secondary clusters, including number of nuclei, cell type annotation, and top marker genes by cluster (Sheets 1-2); number of nuclei assigned to each cluster by subject (Sheets 3-4); and normalized expression values of variable genes (used for clustering) by cluster (Sheets 5-6).

File Name: Supplementary Data 4
Description: Results of ToppFun functional enrichment testing of cluster marker genes. Hypergeometric Test, one-sided, 5% False Discovery Rate adjustment.

File Name: Supplementary Data 5
Description: A priori genes of interest organized by category (ligand, receptor, transcription factor, or other) and molecular system.

File Name: Supplementary Data 6
Description: Sheet 1: List of genes exhibiting IEG-like expression. Sheet 2: Results of linear-mixed effect regression modeling of building-, quivering-, and gonadal-associated IEG expression across clusters and gene-defined cell populations. Additional columns show minimum raw p-value, maximum raw p-value, and 5% FDR-adjusted harmonic mean p-value across models. Final significance call indicated in the "significant" column.

File Name: Supplementary Data 7
Description: Results of linear-mixed effect regression modeling of building-, quivering-, and gonadal-associated gene expression across primary (Sheet 1) and secondary (Sheet 2) clusters. Additional columns show minimum raw p-value, maximum raw p-value, and 5% FDR-adjusted harmonic mean p-value across models. The multiple comparisons adjustment was made for all genes x all clusters.

File Name: Supplementary Data 8
Description: Results of ToppFun functional enrichment testing of genes that exhibited gonadal-, building-, and quivering-associated upregulation. Hypergeometric Test, one-sided, multiple methods for multiple comparisons adjustment are presented as columns and labeled with the correspond method.

File Name: Supplementary Data 9
Description: ERE-containing genes.

File Name: Supplementary Data 10
Description: List of genes that positively regulate neurogenesis.

File Name: Supplementary Data 11
Description: Results of linear-mixed effect regression modeling of building-associated gene expression in radial glia as a whole (Sheet 1) and in radial glial subclusters (Sheet 2). Additional columns show minimum raw p-value, maximum raw p-value, and 5% FDR-adjusted harmonic mean p-value across models. The multiple comparisons adjustment was made for all genes x all subclusters tested.

File Name: Supplementary Data 12
Description: List of genes annotated as markers of radial glial functional states including quiescence, cycling, or neuronal differentiation.

File Name: Supplementary Data 13
Description: Results from testing enrichment of marker genes for radial glial quiescence (Sheet 1), cycling (Sheet 2), and neuronal differentiation (Sheet 3) across radial glial subclusters. Raw and Bonferroni-adjusted p-values are presented. The proportion of permutations that exceeded the observed enrichment result when analyzing random genes expressed at similar levels is also presented.

File Name: Supplementary Data 14
Description: Species list, behavioral phenotype, and accession numbers for comparative genomic analyses.

File Name: Supplementary Data 15
Description: CDG and CDG module list (Sheet 1) and comparative genomics results across 10kb bins (Sheet 2).

File Name: Supplementary Data 16
Description: Results from testing enrichment of CDG enrichment (gene list in Sheet 1) across primary clusters (Sheet 2), secondary clusters (Sheet 3), gene-defined populations across all nuclei (Sheet 4), and gene-defined populations with primary clusters (Sheet 5) and secondary clusters (Sheet 6). Raw and Bonferroni-adjusted p-values are presented. The proportion of permutations that exceeded the observed enrichment result when analyzing random genes expressed at similar levels is also presented.

File Name: Supplementary Data 17
Description: Results from testing enrichment of CDG module enrichment (gene list in Sheet 1) across primary clusters (Sheet 2), secondary clusters (Sheet 3), and radial glial subclusters (Sheet 4). Raw and Bonferroni-adjusted p-values are presented. The proportion of permutations that exceeded the observed enrichment result when analyzing random genes expressed at similar levels is also presented.

File Name: Supplementary Data 18
Description: Correlation of CDG module score with normalized expression levels of transcription factors across radial glia. Pearson's R, raw p-value, and Bonferroni-adjusted p-values are presented. Genes annotated as transcription factors that were not detected in radial glia in our data are shown, with Pearson's R, p-values, and adjusted p-values all shown as "NA".

File Name: Supplementary Data 19
Description: CellChat results showing connection weights among clusters and gene-defined populations. The proportion of permuted connection weights among randomly sampled populations of the same size is presented (raw and Bonferroni-adjusted). Sheet 2 shows these values by subject. Sheet 3 shows ligand-receptor pairs that were important for specific connections.

File Name: Supplementary Data 20
Description: Results of linear mixed-effects regression analysis of building-associated changes in CellChat weights among primary clusters, secondary clusters, build-IEG+ populations, and radial glial subclusters. Beta estimate and raw p-value for the effect of building versus control (tested as a categorical variable), number of models significant (maximum of 6), and the 5% FDR-adjusted harmonic mean p-value are presented for each analyzed sender-receiver pair.

File Name: Supplementary Data 21
Description: Summary of mediation analyses conducted to identify candidate mediators of building-associated effects.
