# Supplementary material for: Cellular profiling of a recently-evolved social behavior in cichlid fishes
Source: Nat Commun. 2023 Aug 14;14:4891. doi: 10.1038/s41467-023-40331-9 (PMC10425353; doi:10.1038/s41467-023-40331-9)
Supplement: Supplementary file 25 — Reporting Summary [file 41467_2023_40331_MOESM25_ESM.pdf]

Corresponding author(s): Jeffrey T. Streelman, Patrick T. McGrath, Zachary V. Johnson

Last updated by author(s): 6-12-23

# Reporting Summary

Nature Portfolio wishes to improve the reproducibility of the work that we publish. This form provides structure for consistency and transparency in reporting. For further information on Nature Portfolio policies, see our Editorial Policies and the Editorial Policy Checklist.

## Statistics

For all statistical analyses, confirm that the following items are present in the figure legend, table legend, main text, or Methods section.

| n/a | Confirmed | |
|---|---|---|
| ☐ | ☒ | The exact sample size (*n*) for each experimental group/condition, given as a discrete number and unit of measurement |
| ☐ | ☒ | A statement on whether measurements were taken from distinct samples or whether the same sample was measured repeatedly |
| ☐ | ☒ | The statistical test(s) used AND whether they are one- or two-sided<br>*Only common tests should be described solely by name; describe more complex techniques in the Methods section.* |
| ☐ | ☒ | A description of all covariates tested |
| ☐ | ☒ | A description of any assumptions or corrections, such as tests of normality and adjustment for multiple comparisons |
| ☐ | ☒ | A full description of the statistical parameters including central tendency (e.g. means) or other basic estimates (e.g. regression coefficient) AND variation (e.g. standard deviation) or associated estimates of uncertainty (e.g. confidence intervals) |
| ☐ | ☒ | For null hypothesis testing, the test statistic (e.g. $F$, $t$, $r$) with confidence intervals, effect sizes, degrees of freedom and $P$ value noted<br>*Give P values as exact values whenever suitable.* |
| ☒ | ☐ | For Bayesian analysis, information on the choice of priors and Markov chain Monte Carlo settings |
| ☐ | ☒ | For hierarchical and complex designs, identification of the appropriate level for tests and full reporting of outcomes |
| ☐ | ☒ | Estimates of effect sizes (e.g. Cohen's $d$, Pearson's $r$), indicating how they were calculated |

*Our web collection on statistics for biologists contains articles on many of the points above.*

## Software and code

Policy information about availability of computer code

| | |
|---|---|
| Data collection | A previously described custom script was used to identify sand change from video data (https://github.com/ptmcgrat/CichlidActionDetection) and a trained 3D ResNet was used to annotate behaviors from video data (https://github.com/ptmcgrat/CichlidActionClassification). Nuclei were sorted using FACSDiva software (BD Biosciences, v8.0.1). DNAsequencing data was processed using Picard tools and GATK (v4.1.8.1), and was matched to nuclei using Demuxlet. CellRanger (10X Genomics, v3.1.0) was used to process raw single nucleus RNA-sequencing data. Raw spatial transcriptomics data was processed using Space Ranger (10X Genomics, v1.3.1). |
| Data analysis | FACS sorting data was visualized using FlowJo (v10.6.0). Single nucleus RNA-sequencing (snRNA-seq) data was analyzed using a variety of R packages, including Seurat (v3.2.2; dimensionality reduction, clustering, marker gene analysis), PROreg (v1.2; gene score analysis, e.g. immediate early genes), glmmseq (v0.5.5; differential gene expression analysis), DESeq2 (v1.38.3; differential gene expression analysis), scran (v1.26.2; size factor calculation), lme4 (v1.1-31; cluster proportion analysis), harmonicmeanp (v3.0, FDR-adjusted p-value estimation across models), qvalue (v2.30.0, q-value calculation), WGCNA (v1.70-3, weighted gene correlation network analysis), cluster (v2.1.0, hierarchical clustering), bruceR (v0.8.9; mediation analysis), mmabig (v3.1.0, mediation analysis). Estrogen response elements were detected using bedtools (v2.29.1). Comparative genomics analyses were performed using vcftools (v0.1.17) and bcftools (v1.11). Spatial transcriptomics data was integrated with snRNA-seq data using Seurat (v4.1.0). Cell-cell communication analysis was performed using CellChat (v1.5.0). Core analysis scripts used in this study are publicly available at https://github.com/streelmanlab/cichlid_sn. |

For manuscripts utilizing custom algorithms or software that are central to the research but not yet described in published literature, software must be made available to editors and reviewers. We strongly encourage code deposition in a community repository (e.g. GitHub). See the Nature Portfolio guidelines for submitting code & software for further information.

## Data

Policy information about availability of data

All manuscripts must include a data availability statement. This statement should provide the following information, where applicable:

- Accession codes, unique identifiers, or web links for publicly available datasets
- A description of any restrictions on data availability
- For clinical datasets or third party data, please ensure that the statement adheres to our policy

The snRNA-seq and spatial transcriptomics data generated in this study are deposited and publicly available in the National Center for Biotechnology Information (NCBI) Gene Expression Omnibus (GEO) under accession code GSE217619 (spatial transcriptomics data are deposited as a SubSeries) [https://www.ncbi.nlm.nih.gov/geo/query/acc.cgi?acc=GSE217619].

The DNA data generated in this study are deposited and publicly available in the NCBI BioProject databank under accession code PRJNA867404 [https://dataview.ncbi.nlm.nih.gov/object/PRJNA867404].

The reference genome used in this study was the Maylandia zebra UMD2a RefSeq assembly, deposited and publicly available in the NCBI BioProject databank under accession code GCF_000238955.4 [https://www.ncbi.nlm.nih.gov/assembly/GCF_000238955.4]

## Human research participants

Policy information about studies involving human research participants and Sex and Gender in Research.

| | |
|---|---|
| Reporting on sex and gender | N/A |
| Population characteristics | N/A |
| Recruitment | N/A |
| Ethics oversight | N/A |

Note that full information on the approval of the study protocol must also be provided in the manuscript.

# Field-specific reporting

Please select the one below that is the best fit for your research. If you are not sure, read the appropriate sections before making your selection.

☒ Life sciences ☐ Behavioural & social sciences ☐ Ecological, evolutionary & environmental sciences

For a reference copy of the document with all sections, see nature.com/documents/nr-reporting-summary-flat.pdf

# Life sciences study design

All studies must disclose on these points even when the disclosure is negative.

| | |
|---|---|
| Sample size | Because behavior-associated single nucleus RNA-sequencing experiments had not previously been conducted in any species, we did not conduct a power analysis to determine sample sizes for this study. Instead, single nucleus RNA-sequencing (n=38 males) sample sizes were selected based on previous behavioral neuroscience and neurogenomics literature in which hundreds of behavior-associated differential gene expression effects were demonstrated in specific brain regions (or whole brain) in a wide range of species spanning insects, teleost fishes, and mammals. Spatial transcriptomics (n=4 males), sample sizes were chosen to collect two biological replicates in each behavioral state (building, not building). |
| Data exclusions | <1% of nuclei were excluded during pre-processing based on low read counts (putative dying nuclei), high mitochondrial gene expression (>5% of detected genes, putative dying nuclei), or high read counts (putative multiplets). |
| Replication | This study included 19 paired behave and control replicates and analysis of behavior-associated effects statistically accounted for variation among biological replicates (individuals, pairs) to improve robustness of results. |
| Randomization | All building and control subjects had all previously built bowers. The only criteria used for building subjects was expression of above-threshold building within the sampling time window, and the only criteria used for control subjects was the absence of above-threshold building during this window. |

| | |
|---|---|
| Blinding | Blinding was not possible in this study, as experimental subjects are collected from tanks in which a bower is present (building) or not (control). Thus the experimental group assignment of the subject is visually apparent upon collection. |

# Reporting for specific materials, systems and methods

We require information from authors about some types of materials, experimental systems and methods used in many studies. Here, indicate whether each material, system or method listed is relevant to your study. If you are not sure if a list item applies to your research, read the appropriate section before selecting a response.

## Materials & experimental systems

| n/a | Involved in the study |
|---|---|
| ☒ | ☐ Antibodies |
| ☒ | ☐ Eukaryotic cell lines |
| ☒ | ☐ Palaeontology and archaeology |
| ☐ | ☒ Animals and other organisms |
| ☒ | ☐ Clinical data |
| ☒ | ☐ Dual use research of concern |

## Methods

| n/a | Involved in the study |
|---|---|
| ☒ | ☐ ChIP-seq |
| ☐ | ☒ Flow cytometry |
| ☒ | ☐ MRI-based neuroimaging |

## Animals and other research organisms

Policy information about studies involving animals; ARRIVE guidelines recommended for reporting animal research, and Sex and Gender in Research

| | |
|---|---|
| Laboratory animals | Mchenga conophoros, aged 6-14 months (post-fertilization) |
| Wild animals | No wild animals were used in this study. |
| Reporting on sex | Findings only apply to males, as bower building is expressed exclusively by males. |
| Field-collected samples | There were no field-collected samples used in this study. |
| Ethics oversight | All experiments were conducted in accordance with the Georgia Institute of Technology Institutional Animal Care and Use Committee guidelines (IACUC, protocol number A100029). |

Note that full information on the approval of the study protocol must also be provided in the manuscript.

## Flow Cytometry

### Plots

Confirm that:

☒ The axis labels state the marker and fluorochrome used (e.g. CD4-FITC).

☒ The axis scales are clearly visible. Include numbers along axes only for bottom left plot of group (a 'group' is an analysis of identical markers).

☒ All plots are contour plots with outliers or pseudocolor plots.

☒ A numerical value for number of cells or percentage (with statistics) is provided.

### Methodology

| | |
|---|---|
| Sample preparation | Telencephala were lysed, centrifuged, and filtered before nuclei were isolated by FACS. |
| Instrument | BD FACSAriaTM Fusion Cell Sorter, BD Biosciences |
| Software | BD FACSDiva (sorting), FlowJo (plotting) |
| Cell population abundance | >99% based on DAPI-fluorescence gating, hemocytometer estimates, and downstream quality control |
| Gating strategy | Nuclei were gated based on shape (side scatter area, width, and height), size (forward scatter area), and DNA content (DAPI area). |

☒ Tick this box to confirm that a figure exemplifying the gating strategy is provided in the Supplementary Information.
